# A stochastic algorithm for accurately predicting path persistence of cells migrating in 3D matrix environments

**DOI:** 10.1371/journal.pone.0207216

**Published:** 2018-11-15

**Authors:** Benjamin Michael Yeoman, Parag Katira

**Affiliations:** 1 Mechanical Engineering Department, San Diego State University, San Diego, CA, United States of America; 2 Department of Bioengineering, University of California San Diego, San Diego, CA, United States of America; 3 Computational Science Research Center, San Diego State University, San Diego, CA, United States of America; Shanghai Jiao Tong University Medical School Affiliated Ruijin Hospital, CHINA

## Abstract

Cell mobility plays a critical role in immune response, wound healing, and the rate of cancer metastasis and tumor progression. Mobility within a three-dimensional (3D) matrix environment can be characterized by the average velocity of cell migration and the persistence length of the path it follows. Computational models that aim to predict cell migration within such 3D environments need to be able predict both of these properties as a function of the various cellular and extra-cellular factors that influence the migration process. A large number of models have been developed to predict the velocity of cell migration driven by cellular protrusions in 3D environments. However, prediction of the persistence of a cell’s path is a more tedious matter, as it requires simulating cells for a long time while they migrate through the model extra-cellular matrix (ECM). This can be a computationally expensive process, and only recently have there been attempts to quantify cell persistence as a function of key cellular or matrix properties. Here, we propose a new stochastic algorithm that can simulate and analyze 3D cell migration occurring over days with a computation time of minutes, opening new possibilities of testing and predicting long-term cell migration behavior as a function of a large variety of cell and matrix properties. In this model, the matrix elements are generated as needed and stochastically based on the biophysical and biochemical properties of the ECM the cell migrates through. This approach significantly reduces the computational resources required to track and calculate cell matrix interactions. Using this algorithm, we predict the effect of various cellular and matrix properties such as cell polarity, cell mechanoactivity, matrix fiber density, matrix stiffness, fiber alignment, and fiber binding site density on path persistence of cellular migration and the mean squared displacement of cells over long periods of time.

## Introduction

Cell migration through three-dimensional (3D) fibrous matrix environments is essential to a number of biological processes including immune response and cancer metastasis [1–4]. Great leaps have been made in understanding the forces that drive the migration of cells through 3D environments [5–7]. Cell migration has been shown to strongly depend on the number of cell-matrix adhesion sites [8], the mechanical activity of the cells [9], the stiffness of the cells [10], the stiffness of the cell nucleus [11], the stiffness of the matrix [12], the porosity of the matrix structure [12], matrix fiber density [8], matrix fiber alignment [13], presence of signaling molecules [14], and the presence of matrix modifying enzymes [15]. Numerous experimental and theoretical studies describe the individual or combined influence these cellular and extracellular factors have on the mechanisms, energetics, and ultimately the rate of cell migration within unique tissue environments [16–18]. However, the story describing cell migration is not complete, and assimilation of how all these concomitant factors influence cell migration within a given environment is necessary for a more comprehensive understanding. While the independent effect of cell-matrix adhesion sites, matrix stiffness, matrix porosity, and the presence of matrix restructuring or degrading enzymes on cell velocity is fairly well understood by now [2], their combined effect on the velocity is hard to estimate and quantify based on available literature. In addition, the individual and combined effects of the above-mentioned parameters on the persistence length of the migrating cell remain poorly characterized. The directional persistence of the cell’s path is crucial in determining how far a cell can migrate within a certain matrix environment in a given amount of time and is as critical as the cell speed in predicting the time scales of key biological processes dependent on cell migration.

The idea that the path persistence of cell migration is an important cell mobility characteristic is not new [19,20]. There are a few results available that quantitatively or qualitatively describe the dependence of persistence length of cells migrating in 3D environments on factors such as cell-matrix binding site density [8], matrix density [21], matrix fiber alignment [13,22], matrix degrading enzymes [21,23], and cell polarity [24]. However, obtaining quantitative results over a range of parameter values for a diverse set of factors, especially for 3D cell migration, is extremely tedious. Firstly, quantifying the path persistence of cells with high reliability requires tracking the cells along a trajectory at least 8 to 10 times their persistence length. For slow moving cells, this can be immensely time-consuming experimentally. Secondly, isolating the effects of the various factors from one another to understand their individual role is a difficult task in biological experiments, as the factors can be interlinked in complex ways. This complication can be solved by the use of computational models that can extract the effect of individual parameters from within a large, interdependent parameter space over a wide range of parameter values [16]. With this in mind, there have been recent attempts to use computational modeling to predict the persistence length of cell migration as a function of multiple cellular and matrix properties. There are two main computational approaches that have been explored for this purpose– 1) a cellular-potts based approach where the elements of the matrix are placed on a 3D orthogonal lattice and the cell navigates through this grid space [23,25] and 2) an off grid model with matrix elements distributed randomly within a 3D space to recreate the microscale architecture of the matrix and the cell navigating through this environment based on its interactions with the matrix elements [26–28]. While a lattice-based model can provide information regarding the influence of various matrix properties such as fiber density, matrix porosity and to some extent fiber alignment, it is not an accurate description of the matrix architecture. The matrix architecture that includes the number of crosslinks per fiber and fiber angle along with fiber density and fiber alignment governs additional matrix properties such as stiffness [29] and influences cell polarity [30]. For this reason, off-lattice models can provide a more accurate and holistic model of cell migration in 3D environments but suffer extensively from the high computing power required to keep track of cell interactions with predefined and randomly oriented matrix elements. This can result in exceedingly long simulation times to obtain reliable persistence length data.

Here, we present an off-lattice model for simulating cell migration in 3D environments, where the matrix elements are not predefined, but stochastically generated to mimic the microscale matrix architecture as required to update the current state of the migrating cell. This reduces the need to compute the cell position and interaction with a large number of predefined matrix elements and allows for rapid simulation of long timescale cell migration trajectories. The model also incorporates a large number of previously developed microscale cell-matrix interaction models and empirical relations between known interacting cell and matrix properties from available literature. Using this multiscale, stochastic simulation approach, we have developed an extremely detailed, versatile and fast simulation platform to predict the migration of cells in 3D environments. We use this platform to predict the path persistence of migrating cells as a function of a variety of individual cell and matrix properties.

## Results

### Follow the fiber model

**Fig 1:**
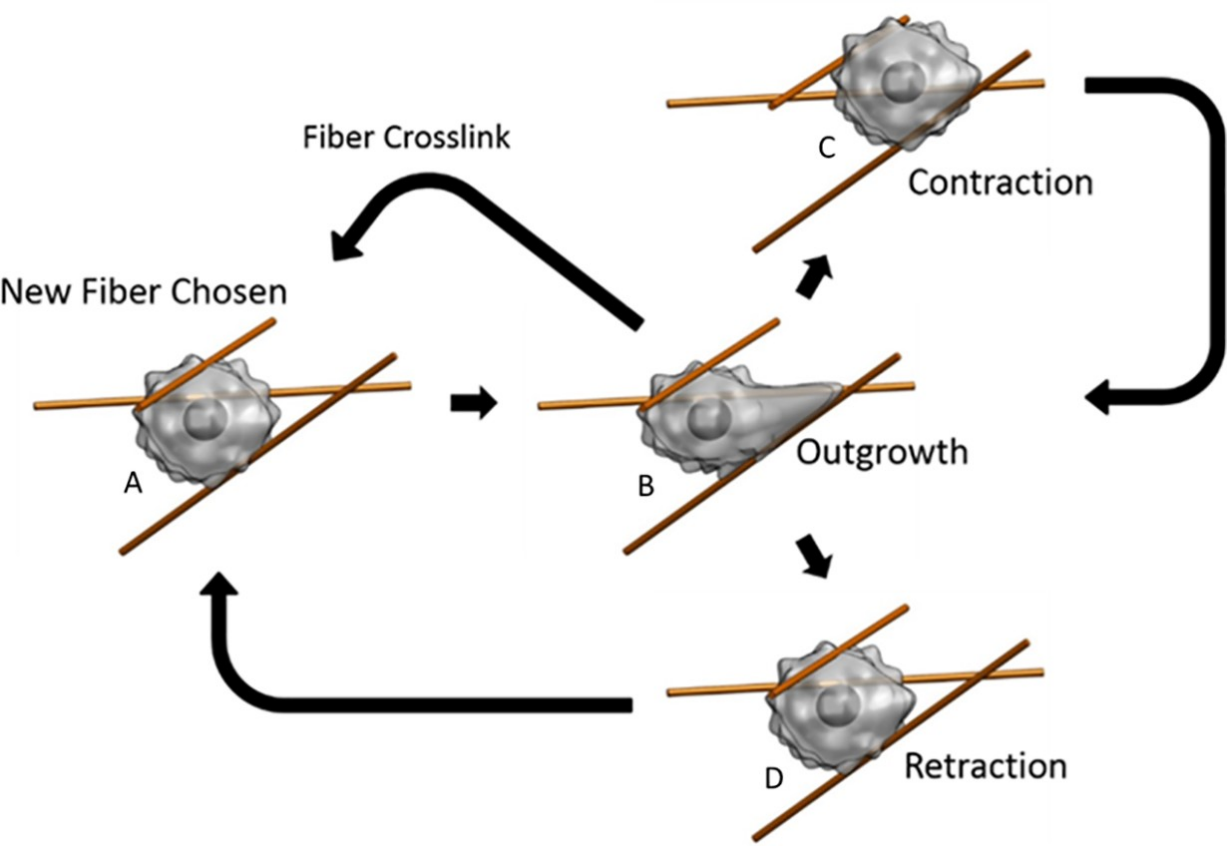
Follow the fiber migration strategy. A) Cell chooses a fiber to crawl along. B) Cell searches for binding sites by extending pseudopod along fiber. If it reaches a crosslink, it follows the intersecting fiber. C) If cell finds enough binding sites it will contract and move along the direction of the extended pseudopod. After contracting, it will continue searching along the same fiber, unless a secondary pseudopod begins to extend along a neighboring fiber. D) If a growing pseudopod does not find enough binding sites along a particular fiber, it will retract and choose a new fiber to search along.

The model describes cell migration using a “follow the fiber” strategy [3] based on the mesenchymal style of migration, summarized in Fig 1. The cell moves by crawling along supporting matrix fibers that act as tracks to guide the cell. With this basic outline, we stochastically recreate how a cell interacts with its environment and gain valuable insight into how changes to the cell and its surroundings affect its overall motility.

At any given stage of this process, the cell exists in one of three distinct phases; outgrowth, retraction, or contraction [27]. The cell enters the outgrowth phase after selecting a single fiber from a set within its immediate surroundings. A pseudopod extends along the chosen fiber in search of ligands distributed along the fiber’s length. If binding sites are too scarce, the cell enters the retraction phase. Here the pseudopod retracts while the cell simultaneously selects a new fiber and extends a new pseudopod along it, reentering the outgrowth phase. Retraction can also occur spontaneously due to the random extension of a new pseudopod, the occurrence of which is based on the cell’s mechanoactivity. Pseudopod contraction causes the contraction phase to occur only when enough bonds form at the pseudopod tip. As the cell contracts, it pulls itself the entire length of the pseudopod at a speed determined from the contractile and friction forces experienced by the cell. Relocation then returns the cell to the outgrowth phase. In this model, we assume proteolytic action takes place at a constant rate during the contraction phase and is parameterized by the viscous friction encountered by the cell during motion. Binding sites along fibers, crosslinks between fibers, and fiber orientation are stochastically generated around the cell as it migrates through 3D space (See methods for further details). The cell’s position and phase are determined at 2 second intervals and the total simulated time is at least 48 hours (S1A–S1D Fig and S2A–S2F Fig).

Using this approach, we are able to rapidly simulate multiple days’ worth of cell migration and generate tens of thousands of cell trajectories in a matter of hours. This allows us to analyze the complex relationships between independent and dependent variables and their overall effect on cell migration. In the following sections, we first validate our model by comparing our results for binding site density and fiber alignment to experimental observations. We then use this model to predict cell speed, path persistence, and mean squared displacement as a function of various cell and matrix properties.

### Effect of binding site density on cell migration

**Fig 2:**
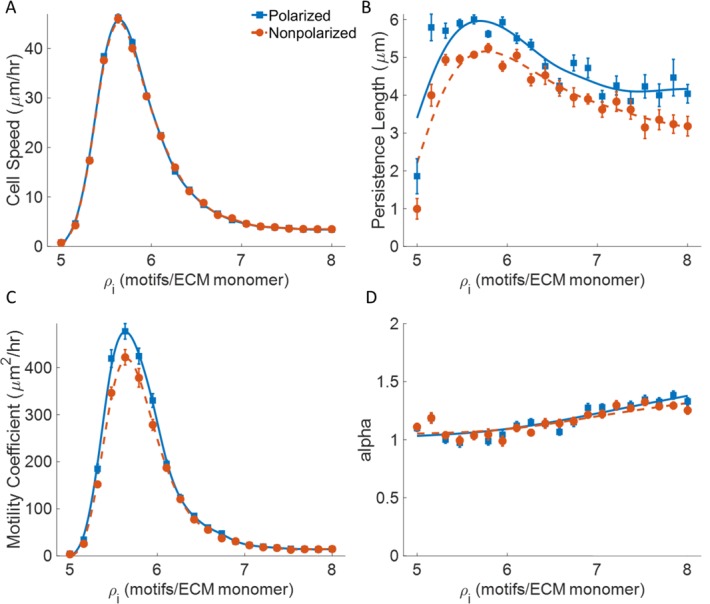
Effect of binding site density. Plots for v_avg_, L_p_, μ, and α as a function of increasing binding site density **A)** Cell speed **B)** Random motility coefficient **C)** Alpha **D)** Persistence length. C_gel_ = 3.7 mg/ml, ρ_fiber_ = 1.0 x 10^−3^ fibers/μm^3^, AI = 0, and t_search_ = 16s. Simulation time = 60 hrs. n = 20. Solid blue lines are polarized cells (◼), dashed red lines are nonpolarized cells (●). Error bars represent ± SEM. Splines added to emphasize trends.

We compare the effect of binding site density predicted in this model to those observed experimentally. In this simulation, binding sites are modeled as the number of individual binding motifs embedded within a single ECM fiber. Fig 2 shows how the binding site density along a fiber affects cell migration, with gel concentration, fiber density, alignment index, and mean pseudopod extension frequency held constant. Binding site density is linearly varied from 5 to 8 binding motifs per fiber monomer. The resulting cell trajectories are analyzed to get cell speed, persistence length, and mean squared displacement as described in the methods section.

We observe a biphasic relationship for cell speed with binding site density (Fig 2A), which matches qualitatively to what has previously been seen experimentally [31]. Our model corroborates the standard explanation for this biphasic trend, where at lower binding site densities the cell often gets stuck searching for suitably large adhesion clusters required for outgrowth and contraction. As binding site density increases and these clusters grow larger, the cell’s velocity decreases as the friction due to binding site dissociation increases. Our model further predicts that the increased binding site density also prevents longer pseudopod extensions, thus decreasing the contraction force generated by the cell [32].

Persistence length also shows a biphasic relationship with binding site density (Fig 2B), which quantitatively matches the experimental data from Burgess *et al*. [8]. They propose that past the optimum cell-substratum adhesion, persistence length begins to decrease as the cell becomes hyper adherent to the substrate at higher binding site densities. This results in competing pseudopods that cause direction changes that bring about the lower persistence. Similarly, our model shows that persistence length is greatest at intermediate binding site densities, where the cell retracts less often and follows the supporting fiber for longer distances (S4 Fig). However, as the binding site density increases, sufficient binding sites for contraction and forward movement can be found readily, thereby decreasing the final extension length of the pseudopod. This increases the total number of pseudopod extensions required to traverse the distance between two neighboring crosslinks along a fiber, in turn increasing the likelihood of a secondary pseudopod extension event before reaching the crosslink and thus lowering the path persistence.

To complement cell speed and persistence length, we also characterize the random motility coefficient, μ, as a measure of cell dispersion over longer periods of time and the exponent, α, as a measure of anomalous diffusion. These values are quantified by fitting a curve to the mean squared displacement described by 〈*R*^2^〉 = *μτ*^*α*^. We find the random motility coefficient matches the experimental data from Burgess *et al*. [8], both qualitatively and quantitatively (Fig 2C). The biphasic dependence of the random motility coefficient on binding site density comes from the contribution of both cell speed and persistence. The densities at which speed and persistence are highest correspond to where the rate of cell dispersion is also greatest. The alpha exponent increases from ~1.0 to 1.3, indicating that the cell’s migration becomes slightly more super-diffusive as binding site density increases (Fig 2D). At lower densities, the cell gets stuck more often, due to a lack of sufficient binding sites along the fiber. Whereas at higher densities, reduction in the number of pseudopod retractions allows for comparatively longer step lengths throughout the cell’s trajectory.

Cell polarity, defined by a preferential pseudopod extension at the front end of the cell, only affects the position of the extending pseudopod on the cell’s membrane. This has no effect on the cell’s instantaneous velocity or the time the cell is stuck at similar binding site densities. This is consistent with experimental observation of 3D cell migration [33]. Persistence length and the random motility coefficient show a slight increase for polarized cells. Cells that maintain their polarity follow a more persistent path due to fewer oscillations and directional changes caused by new pseudopods extending from random regions of the cell membrane. Because instantaneous cell speed is unaffected by cell polarity, the increase in the random motility is solely due to the increased persistence length.

### Effect of fiber alignment on cell migration

To study the effect of fiber alignment, the alignment index is varied so that the angular deviation of fibers along the alignment direction ranges from 20 degrees on average to completely random. Enhanced cell migration due to aligned fibers has been observed experimentally [13,22], and is often found in malignant tumors [22,34]. Our simulations show that a cell polarity induced directional bias is essential for this trend to emerge. Without any such bias the cell simply oscillates back and forth, limiting the cell’s long-term persistence(S5A Fig). A similar behavior has been induced experimentally [24] by the disassembly of microtubules, which are necessary for the regulated distribution of inhibitory signals of protrusions at the trailing end of the cell. With our simulation, we observe oscillations with a similar periodicity between 45–170 minutes in cells with no defined leading edge (S5B and S5C Fig).

**Fig 3:**
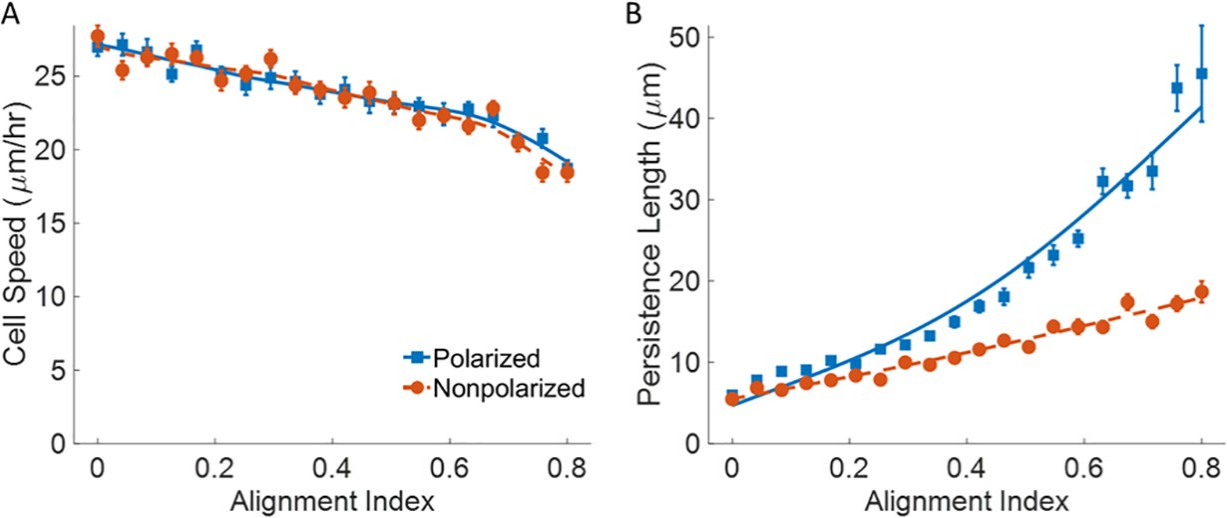
Effect of fiber alignment. Plots for v_avg_ and L_p_ as a function of increasing alignment index **A)** Cell speed **B)** Persistence length. C_gel_ = 3.7 mg/ml, ρ_i_ = 6 sites/monomer, ρ_fiber_ = 1.0 x 10^−3^ fibers/μm^3^, and t_search_ = 16s. Simulation time = 48hrs. n = 20. Solid blue lines are polarized cells (◼), dashed red lines are nonpolarized cells (●). Error bars represent ± SEM.

We further find that fiber alignment predominately affects cell migration efficiency by acting on the persistence length rather than cell speed. Cell speed shows a minor decrease for both polarized and non-polarized cells (Fig 3A), due to a decrease in matrix stiffness with lower crosslink densities. Fiber alignment has the greatest effect on persistence for polarized cells (Fig 3B). As with persistence length, the random motility coefficient was consistently higher for polarized cells (S6A Fig). Alpha was found to increase more for polarized cells, again showing how oscillations affect the cell’s diffusivity (S6B Fig).

### Multi-factor analyses

The main advantage of this model is its ability to simultaneously simulate a wide range of values for several independent factors and analyze their combined effect on cell motility. Here we combine multiple independent variables to predict how they collectively influence cell speed, persistence length, and mean squared displacement.

#### Effect of collagen concentration on cell migration

**Fig 4:**
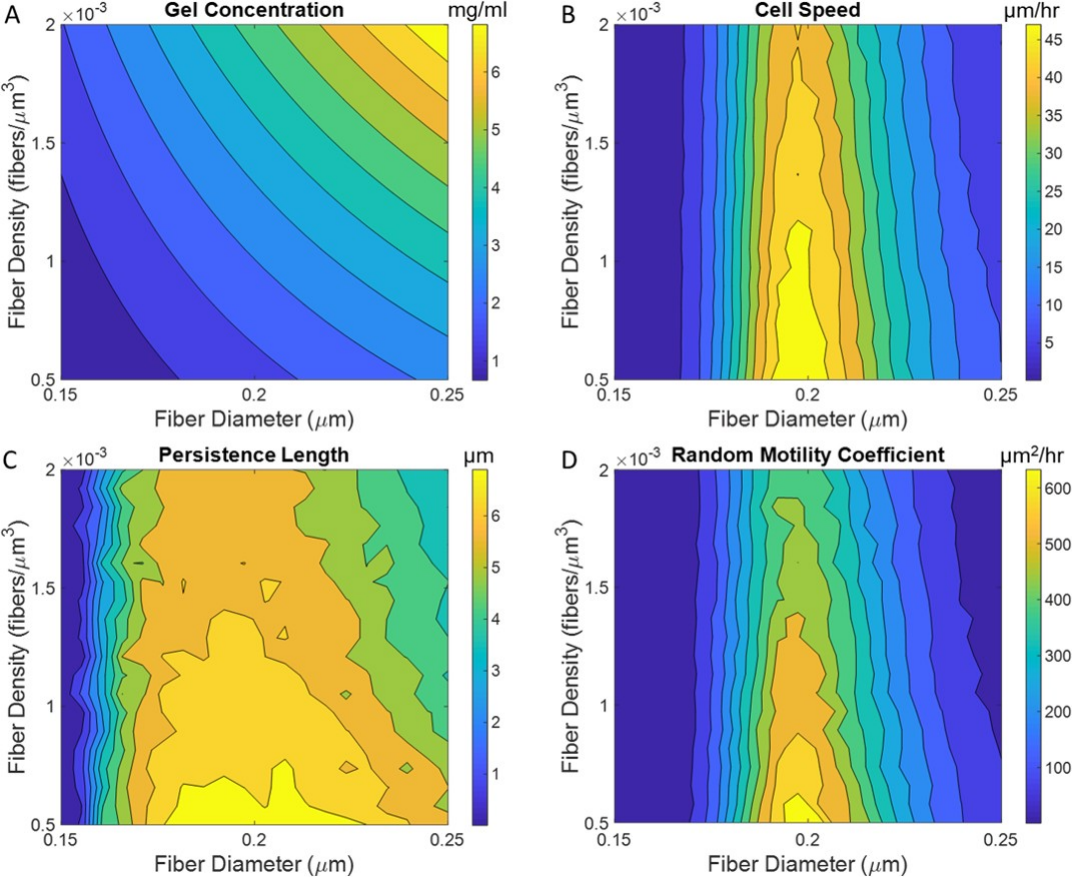
Effect of collagen concentration. Contour plots for gel concentration, v_avg_, L_p_, and μ as a function of increasing fiber diameter and density **A)** Gel concentration **B)** Cell speed **C)** Persistence length **D)** Random motility coefficient. ρ_i_ = 6 sites/monomer, ρ_fiber_ = 1.0 x 10^−3^ fibers/μm^3^, AI = 0, and t_search_ = 16s. Simulation time = 48hrs. n = 20. 10 contour levels automatically chosen by MATLAB are used.

The gel concentration of the collagen matrix is a function of both the mean fiber diameter and fiber density (Eq 5). Experimentally, these two parameters can be independently varied [35–38], but the majority of migration assays only look at the overall effect of gel concentration, rather than the individual role of either fiber diameter or density on cell motility [21,39]. Here we simulate the effect of these two parameters by varying the fiber diameter and density independently, while keeping the gel concentration within 0.5–6 mg/ml, Fig 4A. All other parameters are held constant in this analysis.

The biphasic trend for cell speed emerges as an effect of the fiber diameter, whereas increasing fiber density only results in a minor decrease in cell speed. The strong dependence of the biphasic trend on fiber dimeter is because the increased surface area of the fiber exposes the cell to more binding sites embedded within the collagen monomers, thus increasing the number of bonds the cell forms with the underlying fiber. This biphasic trend with gel concentration has often been seen experimentally in 2D and 3D migration studies [40–42], and has been attributed to the same balance between forward and rearward traction forces as with binding site density. Our model shows that steric hindrances from increased fiber density have significantly less effect on the overall migration. Persistence length and the random motility coefficient also show the same biphasic trend (Fig 4C and 4D) with increasing fiber diameter, which can also be explained by the same effect seen with binding site density.

#### Effect of cell mechanoactivity on cell migration

**Fig 5:**
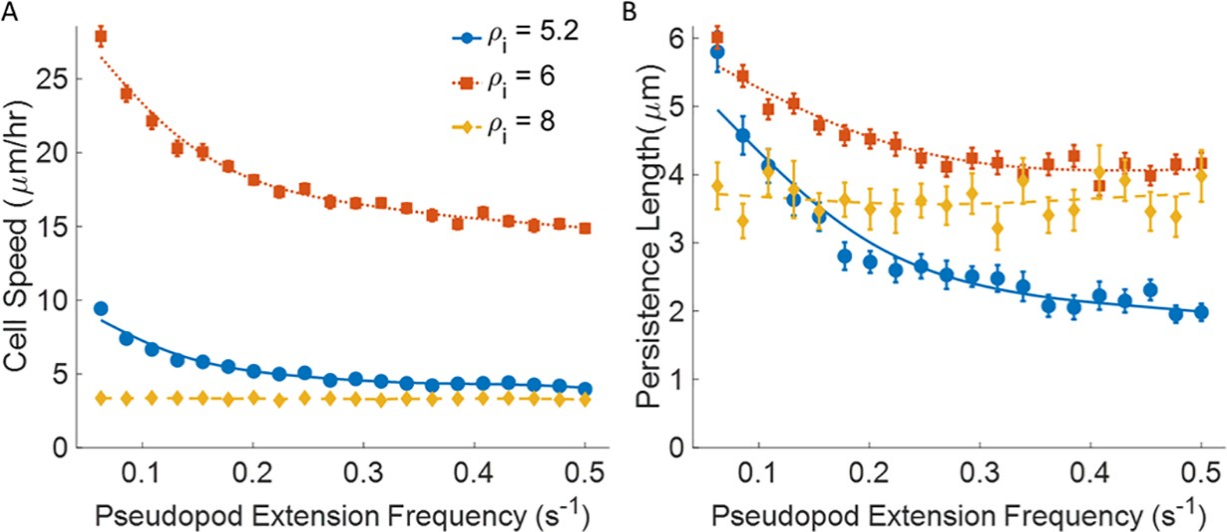
Effect of cell mechanoactivity. Plots for v_avg_, L_p_, μ, and α as a function of increasing cell mechanoactivity **A)** Cell speed **B)** Persistence length. C_gel_ = 3.7 mg/ml, ρ_fiber_ = 1.0 x 10^−3^ fibers/μm^3^, and AI = 0. Simulation time = 48hrs. n = 20. Dotted red lines are 5.2 motifs/monomer (◼), solid blue lines are 6 motifs/monomer (●), dashed yellow lines are 8 motifs/monomer (◆). Error bars represent ± SEM.

We define cell mechanoactivity by the frequency at which the cell spontaneously extends new pseudopods. Here we look at its effect on cell motility at different binding site densities by varying the frequency of pseudopod extensions from 0.06–0.5 s^-1^. Cell speed decreases with increasing extension frequency (Fig 5A), with the greatest changes seen in cells migrating in ECMs with more moderate binding site densities. Because the existing pseudopod retracts as the new one extends, the inverse of pseudopod extension frequency, or the average search time, limits the length to which a pseudopod can grow. These shorter pseudopods exhibit a weaker contractile force [32] (see methods), and instantaneous velocity is reduced. Little change is observed in cell speed for cells migrating in high binding site density ECMs because the higher density already limits the length of the extending pseudopod. The same trend is seen with the random motility coefficient (S7 Fig).

Persistence length is also strongly influenced by the average pseudopod extension length. In less adherent environments, less active cells have the opportunity to fully extend their pseudopods, but as the cell becomes more active, the average extension length decreases. Therefore, we observe that persistence length decreases as the mechanoactivity increases for cells migrating in these environments. At higher densities, the extension length of the pseudopod is already limited, thus providing a relatively flat relationship similar to that of cell speed, Fig 5B. The crossover of blue and yellow lines suggest that higher pseudopod extension frequency is detrimental at lower binding site densities. This may explain the need for the mesenchymal to amoeboid transition seen in some cancer cells. With these cells, amoeboid migration usually prevails in an ECM of low stiffness with few adhesion sites [43,44], and can be brought about by conventional chemotherapy, or treatment with integrin-blocking antibodies or protease inhibitors [44,45]. By switching to amoeboid migration, cell migration is no longer dependent on the binding site density, allowing the cell to maintain its migratory ability.

#### Motility phenotypes in multi-parameter space

**Fig 6:**
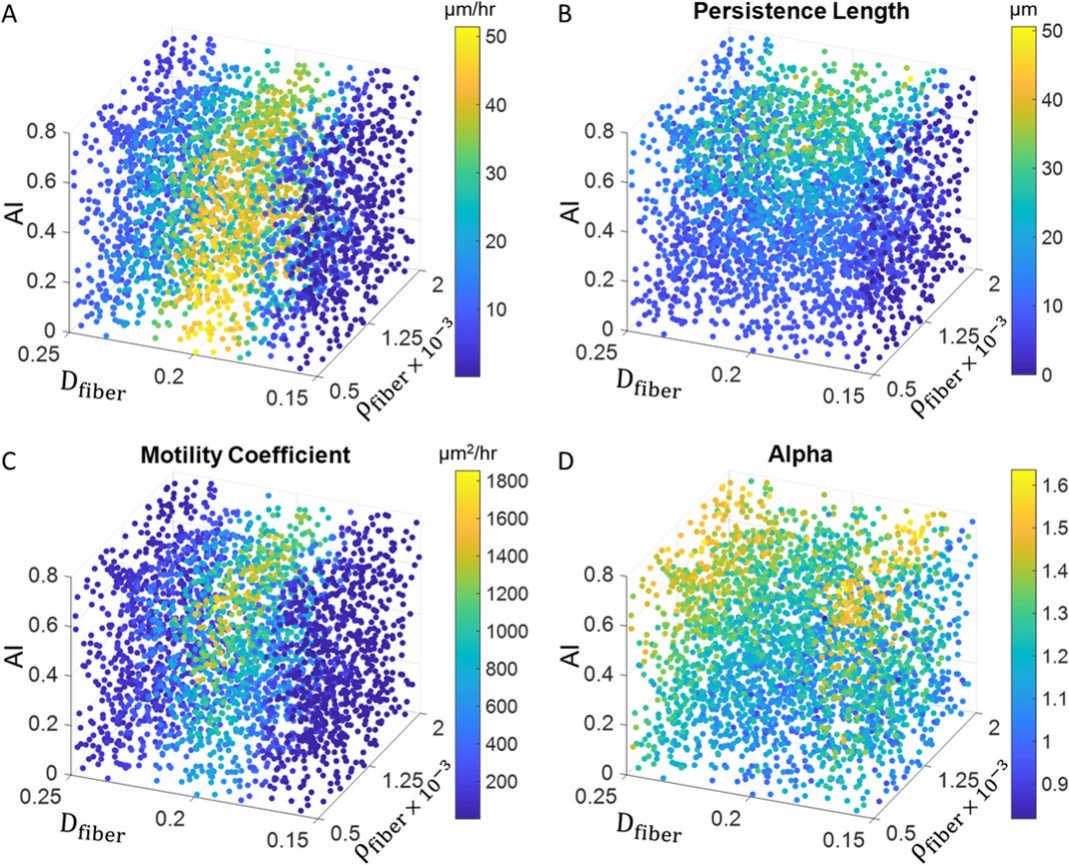
Combined effect of fiber alignment, fiber density, and fiber diameter. **A)** combined effect on cell speed **B)** combined effect on persistence length **C)** combined effect on random motility coefficient **D)** combined effect on alpha. Color bars show corresponding value for each parameter. Each point is the average of 10 separate simulations.

Fig 6 illustrates the combined effect of fiber alignment, fiber density, and fiber diameter on cell speed, persistence length, random motility coefficient, and alpha. The scatter plots are obtained from using 3,000 simulated cells, each with a different set of randomized initial conditions. Each point represents the average on 10 separate simulations and colored according to its value. Simulations for all 30,000 trajectories were performed in parallel and completed in less than 4 hours. While these plots highlight the trends seen above, they provide greater insight into how these trends may change with the additional influence of other parameters. This analysis also underlines the main advantage of our model, in that it can rapidly simulate tens of thousands of cells in a matter of hours to predict the combined effect of multiple variables on each measure of motility.

**Fig 7:**
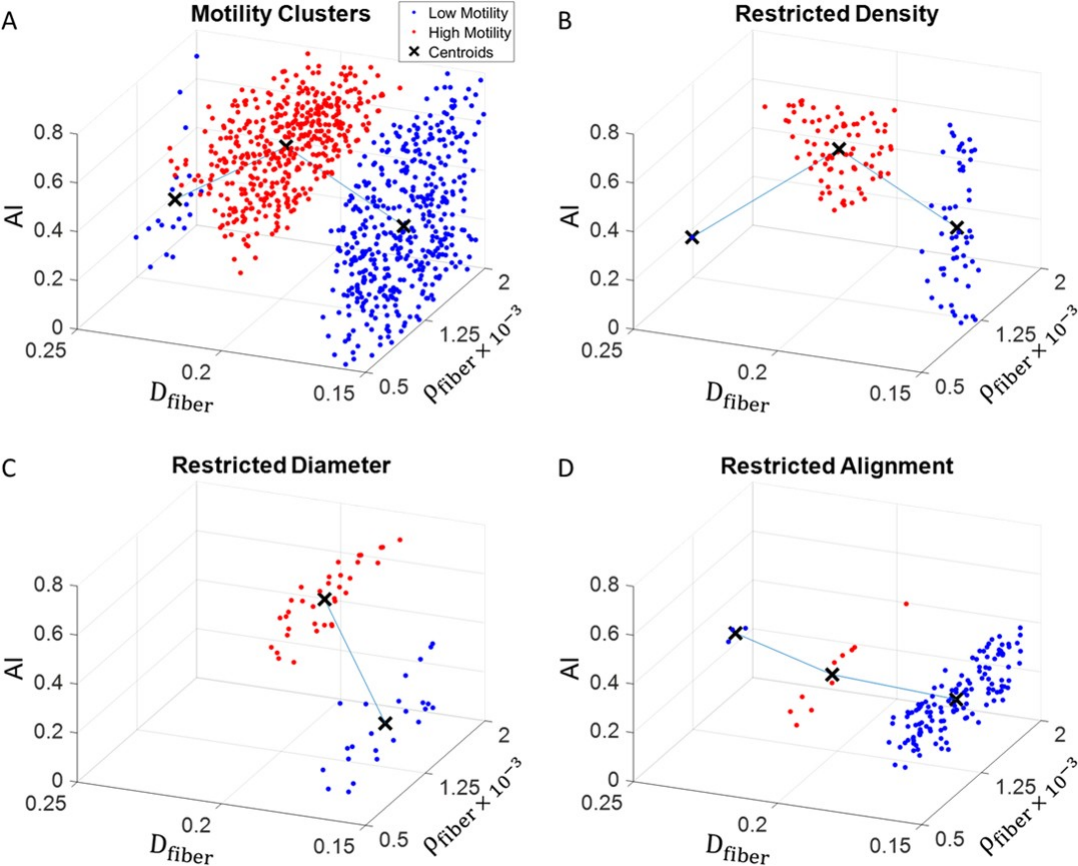
Cluster classification of high and low states of diffusion. Red spheres represent top 85% and bottom 15% of diffusivity. **A)** diffusivity clusters over entire range of fiber alignment, fiber density, and fiber alignment **B)** diffusivity clusters with restricted fiber density **C)** diffusivity clusters with restricted fiber diameter **D)** diffusivity clusters with restricted fiber alignment. Each point is the average of 10 separate simulations.

We then classify each point as either high or low motility based on the product of cell speed and persistence length, and group each set into clusters. In Fig 7, it is visualized with only 3 parameters in 3D, but this technique can be extended to any number of parameters to be used in the analysis. The blue points represent the cells in the top 85% of motility and the red points represent the bottom 15% of motility. The line connecting the centroids of the separate clusters indicates the fastest route to switch between high and low motility states, and its slope specifies how much each parameter should be adjusted. Fig 7C and 7D show the fastest route to switching motility phenotype when one of the parameters is restricted to a narrow range of values. This information can be extremely valuable for identifying cellular or extra-cellular targets to rapidly alter cell motility in the presence of constraints on the degree to which biophysical parameters can be controlled. This can also provide an estimate on the potential approach cells might take in order to transition from one motility phenotype to another. The results in Fig 7 are specific to the other parameter values held constant in these simulations and are only representative of a mesenchymal migration process. However, this tool can be applied to actual experimental and clinical scenarios to better understand the interplay between multiple parameters and help determine the smallest alterations necessary to transition between motility states.

### Incorporating temporal changes in matrix architecture

**Fig 8:**
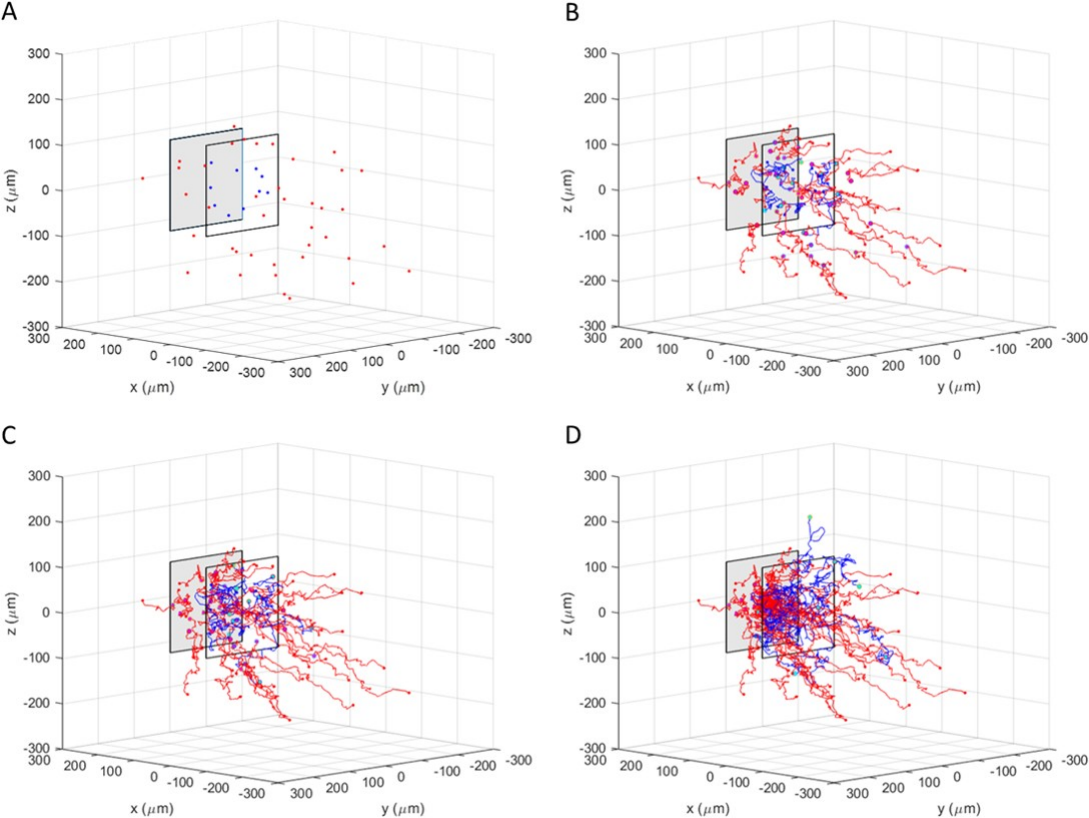
Cell-cell interaction. **A)** Initial cell position **B)** Cell position after 3 hrs **C)** Cell position after 6 hrs **D)** Cell position after 12 hrs. Red cells are attracted to the gray square; blue cells are inert to signal.

This model can be adapted to simulate dynamic environments with multiple cell types. We demonstrate this by simulating a peculiar scenario—the migration of one cell type towards a stimulus, inadvertently reorganizing the surrounding matrix and causing a second cell type that is inert to the stimulus to migrate away from the origin of the stimulus. In this example, both cell types have the same mechanical interactions with the ECM, while only one of them responds to the external stimulus by increasing its mechanoactivity towards the source of the stimulus. Initially, the cells are spatially distributed as shown in Fig 8A, with the stimuli responsive cells distributed evenly throughout the matrix, and the stimuli inert cells (blue) clustered in a plane 100 μm away from the stimulus. As both these cell types migrate through the matrix, they modify the local environment by degrading matrix fibers and reducing the surrounding stiffness. Fig 8A–8D show how the stimuli responsive cells migrate towards the stimulus, altering the matrix along their path and driving the inert cells to migrate from the softened matrix into the surrounding stiffer regions.

## Discussion

### Predictions of cell motility

We have described above how the speed and persistence of cell migration varies as a function of binding site density, fiber alignment, gel concentration, cell mechanoactivity, cell polarity, and the combined effects of fiber alignment, fiber diameter, and fiber density. Moreover, we believe this is the first statistically reliable prediction of cell persistence length for cell migration within physiologically relevant matrix environments (cell and matrix properties summarized in Table 1). It is important to note that the cell motility trends shown here are limited to a follow-the-fiber cell migration strategy within fibrous matrix environments. Additionally only a small subset of parameter values are tested. Hence the trends generated cannot be generalized for all cell migratory behaviors. However, the equations or empirical relations we use to govern the interdependence of the various factors on one another, as well as the rules for pseudopod extension, retraction, or contraction are based on detailed theoretical formulations or experimental observations described in literature. If additional evidence for more detailed mechanisms of pseudopod driven motility is discovered in the future, the model can be modified to account for the altered rules by making only minor changes to the algorithm.

**Table 1:** Model parameters.

Parameter	Definition	Value	Sources
*A*_*cell*_	Surface area of the cell (μm^2^)	Calculated	Ellipsoid Geometry
*A*_*pseudo*_	Area of the pseudopod in contact with binding zone of local fiber (μm^2^)	~0.3	[53,54]
*AI*	Fiber alignment index	0–0.99	[55]
*D*_*fiber*_	Diameter of ECM fiber (μm)	0.05–0.435	[56]
*F*_0_	Adhesion force for given *n*_*b*_ (nN)	Calculated	Eq 1
*F*_*max*_	Maximum force a cell can exert on its surroundings (nN)	0.01–10	[57–59]
*k*_*b*_	Boltzmann’s constant (m^2^ kg s^-2^ K)	1.38 x 10^−23^	
*k*_*ecm*_	Stiffness of the ECM (N/μm)	0.1 x 10^−10^–1 x 10^−10^ in vitro 0.1 x 10^−7^–10 x 10^−10^ in vivo	[42,60,61]
*k*_*gel*_	Gel Stiffness (Pa)	30–6000	[29]
*k*_*I*_	Stiffness of cell-ECM bond (nN μm^-1^)	~0.25 x 10^−9^	[62]
*k*_*off*_	Cell-ECM unbinding rate under zero force conditions (s^-1^)	0.1–100	[61,63,64]
*k*_*on*_	Cell-ECM binding rate (μm^2^/s)	10^−5^–10^−3^	[65,66]
*L*_*fiber*_	Length of ECM fiber (μm)	> 10	[56]
*n*_*x*_	Crosslink multiplier (crosslinks/fiber)	2.09–4.439	[29]
*s*	Cell sphericity (ratio of an ellipsoidal cell’s minor to major axis)	0.1–1	[67]
*T*	Temperature (K)	310	
*t*_*search*_	Average time a pseudopod will extend before retracting and a new one begins extending (s)	2–16	[9,68]
*V*_*cell*_	Volume of the cell (μm^3^)	1000–2000	[69]
*v*_*pseudo*_	Pseudopod extension velocity (μm/s)	~0.45	[9]
η	ECM viscosity (nN s μm^-2^)	~10^−10^	[60]
*θ*_*a*_	Average orientation angle of all fibers within the matrix (degrees)	0	
n12	Cell-ECM bond density at which the generated force is half of *F*_*max*_	100	[61]
*ρ*_*fiber*_	Fiber density of the ECM (fibers/μm^3^)	> 0.002	[70]
*ρ*_*gel*_	Gel density for a given matrix (based on *D*_*fiber*_ and *ρ*_*fiber*_) (mg/mL)	0.5–6	[21,22,27,55]
*ρ*_*i*_	Binding site density (motifs/ECM monomer)	0–25	[8]
*ρ*_*max*_	Number of cell-ECM binding sites needed for pseudopod contraction	150–500	[27]
*ρ*_*min*_	Minimum number of cell-ECM binding sites required for cell to extend pseudopod	50–250	[27]
*ρ*_*X*_	Crosslink density (crosslink/μm^3^)	Calculated	
*ρ*_*Xmax*_	Maximum crosslinks/fiber when fibers are aligned randomly	4.439	[29]
*σ*_*f*_	Standard deviation of the average angle between fibers for a given AI (degrees)	0–70	[55]
*ϕ*_*p*_	Polarity angle of the cell (degrees)	Calculated	

### Application to experimental analyses

The strength of the algorithm described here lies in its ability to simulate cell migration scenarios occurring over large timescales with little computational cost. To couple this model with specific experimental observations, one must identify the appropriate experimental parameter space and relevant cell-matrix interaction rules specific to the cell type being studied. It is possible that measuring all the various cellular and matrix properties within a given experiment is unfeasible. In these cases, the low computational cost of the model makes it possible to simulate a large variety of scenarios to find the parameter combinations that provide the best fit between simulated and experimentally observed motility phenotype. This can then be used to provide insight regarding specific cellular and extracellular properties as well as governing cell-matrix interactions. Additionally, the model can then be used to determine long-term cell migration behavior (difficult to track experimentally) within that experimental/clinical setup, as well as identify targeted modifications of cell and matrix properties to alter cell motility towards a desired phenotype.

### Applications to treatment and motility manipulation

The large input parameter space that can be accounted for during these simulations makes the model extremely well suited to study the combined effect of various cellular and extra-cellular matrix properties in a controlled manner. An example of this is shown in Fig 6 where a 3 parameter space exploring various matrix properties is analyzed for cell migration behavior. Fig 7 shows how this analysis can be used to determine the best strategies of altering matrix properties to change the cell migration behavior. A similar analysis can be performed on an n-parameter space including cellular and matrix mechanical properties, providing insight into how these properties should be managed *in vivo* using existing methods to remodel or disrupt the ECM structure[46–49] or modify cellular polarization [24], integrin expression [46,50], or pseudopod protrusion frequency [51]. This versatility, incorporation of many possible cell-matrix interaction parameters, and the ability to rapidly simulate long-term cell migration for a large number of cells are the key strengths of the proposed algorithm and the described simulation platform. Parameter sweeps for upwards of 50,000 different combinations and generation of trajectories of individual cells can be run in parallel on computing clusters within a matter of hours. This ability to simulate cell migration in a diverse set of environments is of great significance in studying biological processes such as cancer cell metastasis and will help illuminate new targets and strategies for suppressing the migration of metastatic cancer cells.

### Future work

To summarize, we present an off-grid computational model that can simulate cell migration over long time frames to accurately predict path persistence for cell migration in 3D environments. The defining feature of this model is that the matrix environment through which the cell migrates is stochastically generated as needed to update the migration state of the cell (outgrowth, retraction, or contraction). That being said, one of the limitations of the model is that the stochastic nature of the model does not allow for the cell to have a detailed memory of its previous interactions with the matrix elements. This makes it difficult to analyze in detail the cell driven remodeling of the surrounding matrix, and its subsequent effect on secondary cells passing through the same space. We have explored a potential way to overcome this limitation as described in the ‘incorporating temporal changes in matrix architecture’ section, where we coarse grain the influence of individual cells migrating through a region by changing the average mechanical properties of that region over time. However, for future applications, a more detailed change in matrix architecture can be incorporated and updated as cells pass through a given localized region. For example, fiber density or alignment index can be made functions of the spatial coordinates and these functions can be updated as cells migrate through these regions. Also, in its current form, the simulations do not account for direct cell-cell interactions. However, since the model simulates the state of each cell within the same time instant, if two or more cells come within interaction range during a given instant, their states can be updated accordingly.

We are currently working to incorporate various biophysical and biochemical intra- and intercellular interactions within the current framework and aim for the future versions of the algorithm to account for cell-cell interactions, mesenchymal to amoeboid transitioning, cell death and division, and collective migration. Diffusion of signaling molecules that are known to govern the mechanoactivity, integrin expression, or polarity of the cell can be incorporated using a finite difference method to track local concentrations and adjust the cellular response accordingly. Likewise, activation and deactivation of intra-cellular signaling pathways could be implemented in a similar manner; however, since it is the mechanical cell-cell and cell-ECM interactions that underlie migration in our model, only the downstream phenotypic changes to the cell would need to be added. Amoeboid migration can occur in an ECM of low stiffness with few adhesion sites [43,44], and may be brought about by conventional chemotherapy, or treatment with integrin-blocking antibodies or protease inhibitors [44,45]. Changes to the model to account for this remain to be made, but could be added by disregarding the follow-the-fiber scheme, stochastically generating a bleb of an average length in a random direction, and, finally, assigning a force exerted by the cell on the matrix of sufficient magnitude required to squeeze through a stochastically generated pore of random size in the direction of the matrix. While these adaptions to the model are certainly feasible, more experimental evidence is necessary to test the accuracy of their addition.

## Methods

The central algorithm of our simulation is inherently based on the five-step process of mesenchymal cell migration [52]. We use these concepts to build a rule-based simulation that determines what stage of the process the cell is in and when, where, and how fast it will move within a given time step. Several of the underlying factors governing this process are determined stochastically from their corresponding probability density functions. For example, fiber diameter, ligand distribution, and pseudopod protrusion frequency are some of the factors generated stochastically. In this section, we will discuss how these parameters are determined, along with the construction of the migration algorithm.

### Stochastic migration algorithm

**Fig 9:**
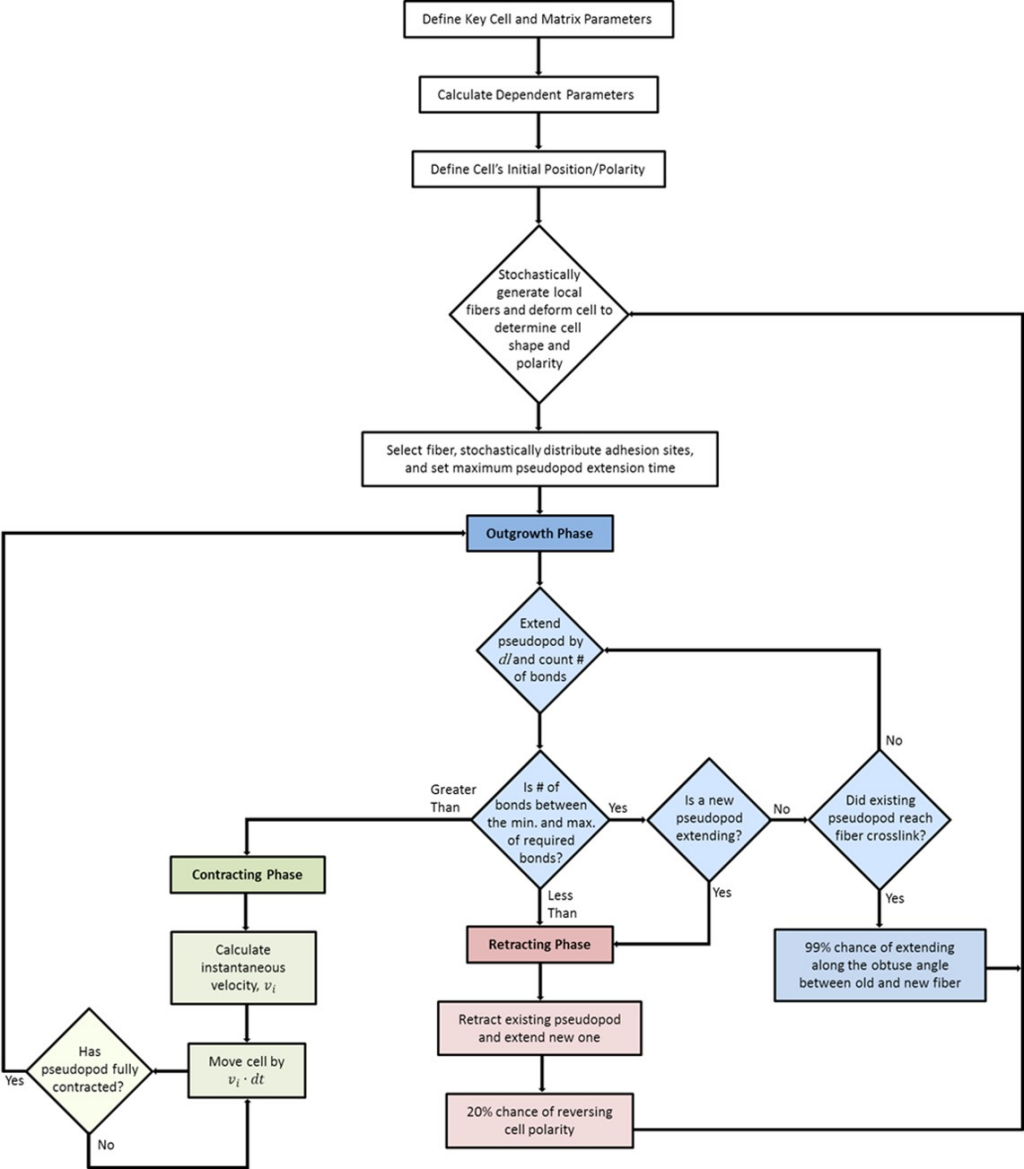
Flow chart for migration algorithm. Blue boxes represent the outgrowth phase, green boxes represent the contracting phase, and red boxes represent the retracting phase.

The flow chart for our simulation process is presented in Fig 9. In the chart, blue boxes represent the outgrowth phase, green boxes represent the contracting phase, and red boxes represent the retracting phase. The algorithm begins by defining the constant cell and matrix parameters, calculating dependent variables, and setting the cell’s initial position and polarity. After these initial conditions are set, the algorithm loops through the outgrowth and contraction or retraction phases described previously. Each loop of the process represents a single simulation time step and corresponds to a predefined period of actual time. The whole sequence is iterated until the specified simulated time is reached, and then cell speed, persistence length, and mean squared displacement are calculated from the cell’s trajectory. The major benefit of this design is that each module of the three phases can easily be altered to account for specific cell behavior or new experimental evidence. With this setup, we can analyze the combined or individual influence of the input parameters on overall motility for almost any cell type in any tissue as long as their specific attributes are known.

### Defining cell and matrix properties

To analyze the cell’s migration characteristics, we vary binding site density, gel concentration, pseudopod extension frequency, and fiber alignment within the range of their physiological values, see Table 1. The methods used to describe the dependent and independent parameters of our model are discussed in the following sections.

#### Total number of binding sites between an extending pseudopod and a matrix fiber

The binding sites are modeled as binding motifs within the individual fibrils that make up the matrix fibers. The simulated fibers are divided up into discrete elements of 10 nm in length *dl*, into which the binding sites are distributed. To account for the reversible association-dissociation kinetics, we use a model developed by Litvinov *et al*. to define the probability that a bond will be formed between the integrin receptor on the cell and the binding sites on the fibril [27]. They came up with the following equation based on experimental data,

$$P_{b}=\frac{k_{on}d_{max}}{k_{on}d_{max}+k_{off}}(1-\exp\left[-(k_{on}d_{max}+k_{off})T\right]$$ (1)

where *k*_*on*_ is the association rate, *k*_*off*_ is the dissociation rate, *d*_*max*_ is the maximum of the receptor or ligand surface density, and *T* is the contact time between the ligand and the receptor. Here, we define the contact time to be length of the pseudopod tip divided by the velocity at which the pseudopod extends. For the range of parameters defined in the simulations, *P*_*b*_ is approximately equal to $\frac{k_{on}d_{max}}{k_{on}d_{max}+k_{off}}$.

Because the cell can only bind to surface peptides, we calculate the average number of binding sites within the surface area of a given fiber element. We assume the pseudopod touches half the surface of a cylindrical fiber. The average number of binding sites within each discrete element *dl* of the fiber probed by the extending pseudopod is then given by

$$n_{b}=\frac{P_{b}\eta_{m}\eta_{f}\rho_{I}D_{f}\pi }{2L_{t}D_{t}}dl$$ (2)

where *η*_*m*_ is the monomer packing fraction within each fibril and *η*_*f*_ fibril packing fraction within each fiber, assumed to be 0.9 and 0.7 respectively. *ρ*_*I*_ is the number of binding sites per fibril monomer, *D*_*f*_ is the fiber diameter generated randomly from the mean diameter based on the normal distribution with a standard deviation of 0.02 μm, and *L*_*t*_ and *D*_*t*_ are the length and diameter of a fibril monomer respectively. The exact number of binding sites in each fiber element probed by the pseudopod tip is then determined stochastically from the Poisson distribution, with the mean of the distribution set equal to *n*_*b*_.

#### Matrix fiber alignment

**Fig 10:**
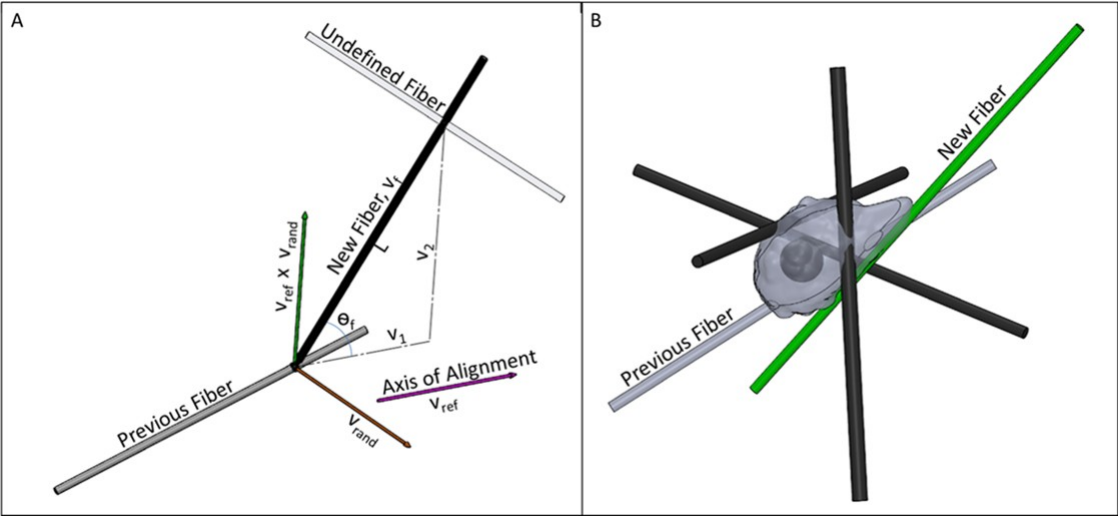
Stochastic fiber generation and selection. **A)** New fiber selection shown for fiber selection in event of a crosslink. The new fiber’s direction is determined from its length L, the angle *θ*_*f*_ it makes with the axis of alignment *v*_*ref*_, and the cross product between *v*_*ref*_ and *v*_*rand*_. **B)** In the event of a new pseudopod, multiple fibers (black and green) are stochastically generated around the cell. The cell then selects the fiber (green) that most closely follows a defined signal gradient or the cell’s extension vector (see pseudopod extension direction section).

An alignment index is used to describe how aligned each fiber is with a default axis of alignment; where when AI = 0 the fibers are completely randomized, and when AI = 1 they are parallel [55]. Except for when AI is zero, the angle each fiber makes with the reference axis is assumed to follow a Gaussian distribution, with the standard deviation *σ*_*f*_ of the fibers’ angle calculated by

$$\sigma_{f}=\sqrt{-1642ln(AI)}$$ (3)

where AI is the alignment index. Each time a new fiber is generated, a random angle *θ*_*f*_ is generated using the normal distribution with *σ*_*f*_ as the standard deviation around a mean angle of 0 degrees with the axis of alignment. The direction vector of the fiber is then determined from *θ*_*f*_, a reference vector parallel to the axis of alignment *v*_*ref*_, and the distance between fiber crosslinks *L*, illustrated in Fig 10A. *L* is defined by a random number generated from the exponential distribution, where $\frac{1}{\sqrt[{3}]{\rho_{X}}}$ is the mean length determined from the crosslink density, *ρ*_*X*_. The following equation is then used to calculate the fiber’s unit vector,

$$v_{f}=\frac{v_{1}+v_{2}}{\left\|v_{1}+v_{2}\right\|}$$ (4)

where $v_{1}=Lcos(\theta_{f})\frac{v_{ref}}{\left\|v_{ref}\right\|}$ and $v_{2}=Lsin(\theta_{f})\frac{v_{ref}\times v_{rand}}{\left\|v_{ref}\times v_{rand}\right\|}$. *v_rand_* is a randomly generated vector used to compute the y and z components of *v*_2_. When AI is defined as zero, the components of the direction vectors for the fibers are simply generated randomly using a uniform random number generator.

New fibers are only generated when a cell retracts a pseudopod or reaches a crosslink. After retraction, a new fiber is selected from a number of randomly generated fibers based on the direction of an external chemical signal or the cell’s polarity and sphericity, Fig 10B. The number of randomly generated fibers at this point is determined from a Poisson distribution, with the mean set equal to the fiber density multiplied by the cell’s volume, while their angle distribution is as described above. The cell is in contact with this set of fibers, and after retracting an existing pseudopod, it can select any fiber in this set to extend a new pseudopod along. At a crosslink, only one new fiber is generated for the cell’s extending pseudopod to follow along.

#### Gel concentration

Cell migration assays are typically made using a collagen scaffold comprised of collagen I hydrogel of varying concentrations [22,55,71]. In our simulation, we vary gel concentration as a function of fiber diameter and fiber density so that it falls within the ranges of those used in experimental cell migration studies [21,22,27,55]. The matrix in our simulation represents type I collagen fibers, consisting of hierarchical levels of monomers and fibrils bundled together to form thicker fibers. The given gel concentration is calculated from the average size of the surrounding fibers, which is determined from the molecular weight of tropocollagen and an assumed monomer packing fraction (*η*_*m*_) of 0.9 and fibril packing fraction (*η*_*f*_) of 0.7. This conversion can be reduced to the following equation,

$$C_{g}=\frac{D_{f}\eta_{m}\eta_{f}\rho_{F}{m_{t}L}_{F}}{{D_{t}}^{2}(L_{t}+0.067)(6.022\times 10^{8})}$$ (5)

where *D*_*f*_ is the fiber diameter, *D*_*t*_ is the diameter of a single tropocollagen, *ρ*_*F*_ is the fiber density, *L*_*F*_ is the average length of an individual fiber, and *m*_*t*_ is the molecular mass of a tropocollagen. This equation also accounts for the 67 nm gap between the ends of tropocollagen molecules [56]. The diameter of fibers in a given matrix is assumed to be normally distributed, and we use this mean value to stochastically set the diameter of the cell’s selected fiber.

Other fibrillar matrix elements, such as fibronectin or laminin, may be used in place of collagen by modifying the relevant parameter values (i.e. stiffness, diameter, length, crosslink density, etc.). Likewise, synthetic polymers that form fibrillar structures can also be used, as long as they can be functionalized with binding sites. Matrices composed of a mixture of fibrillar elements such as Matrigel would require additional variability in the stochastic generation of fibers contacting the cell. The follow the fiber strategy currently employed in this model does not allow for matrices that are not comprised of fiber elements, but could be modified to simply have the cell search random directions for binding sites stochastically distributed throughout the matrix.

#### Pseudopod extension frequency

Pseudopod extension frequency relates to the mechanoactivity of the cell and is defined by the inverse of the average time between pseudopod extensions. In our model, we vary this time from 2 to 16 seconds [9,68]. Every time a new pseudopod begins extending, the existing pseudopod begins to retract [72] and the cell searches for binding sites along a new fiber using the extending pseudopod. The time at which a new pseudopod begins to extend is set by a random number generated from an exponential random distribution with the given mean time between pseudopod extensions. The time between extensions limits the length that a pseudopod can grow, with a maximum allowable time of 32 seconds. With the pseudopod extension velocity set to 0.45 μm/s, this effectively limits the extension length to 14.4 μm. This time is tracked from the beginning of the extension of a new pseudopod and is incremented every time step the cell is in the outgrowth phase. It is then reset any time a new pseudopod begins extending due to retraction or contraction.

#### Cell sphericity

**Fig 11:**
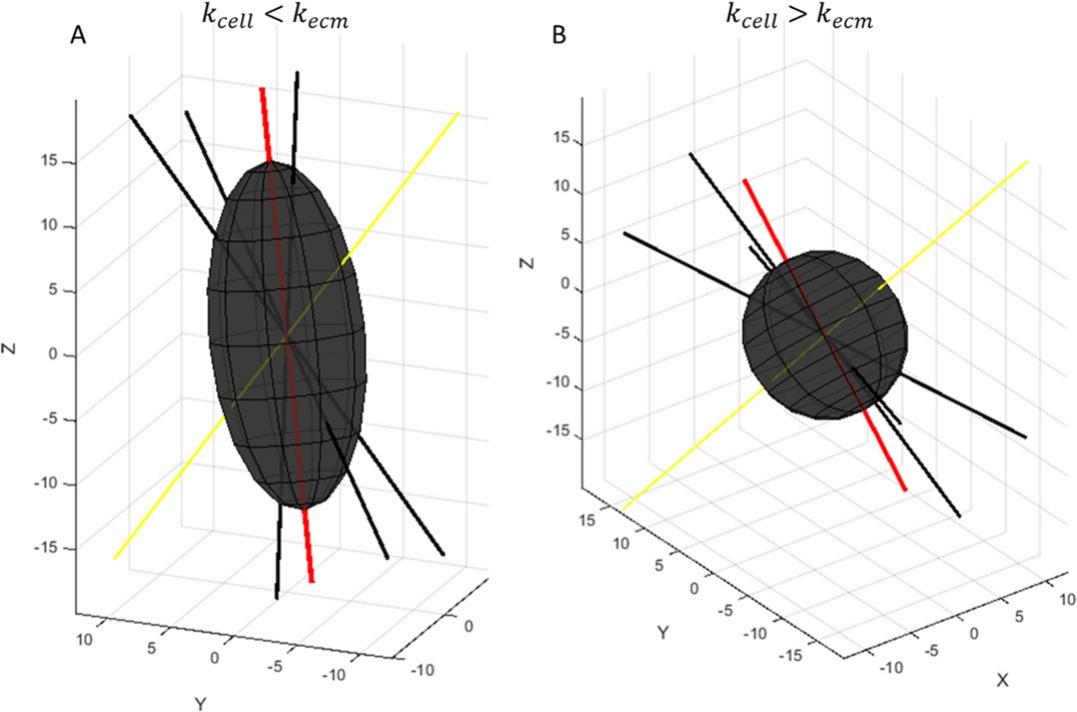
Cell sphericity. **A)** deformation of soft cell in stiff matrix **B**) deformation of a stiff cell in a soft matrix. The yellow line represents the cells previous fiber, the black lines are the surrounding fibers, and the red line is the elongation vector, *v*_*e*_.

To calculate the cell’s sphericity, we assume it is dependent on the direction of the fibers surrounding the cell, the number of bonds between the cell and the surrounding fibers, and the stiffness of both the cell and the ECM, Fig 11. To simplify the deformation of the cell, the direction in which the cell is elongated is defined by the average direction of each of the fibers touching the cell. This elongation vector is then used to define the direction of the cell’s major axis and subsequently its front and rear poles.

The cell is modeled as a prolate or oblate ellipsoid depending on the alignment of the surrounding fibers. The angles between the elongation vector and the surrounding fibers determine the ratio between the major and minor axes of the ellipsoid, which is defined by the following equation,

$$\frac{a_{0}}{b_{0}}=\frac{\sum_{i=1}^{n}cos(\theta_{i})}{\sum_{i=1}^{n}sin(\theta_{i})}$$ (6)

where $\frac{a_{0}}{b_{0}}$ is the ratio between the cell’s major and minor axes, *θ*_*i*_ is the angle between the elongation vector and each surrounding fiber, and *n* is the number of surrounding fibers. a_0_ and b_0_ are then corrected to account for binding site strength and both cell and ECM stiffness. Assuming the volume of the cell remains constant, the length of the cell’s major and minor axis can be calculated using the following equations,

$$a={\left(\frac{3V_{cell}}{4\pi }\frac{\left(k_{cell}+k_{eq}\right)}{(\frac{a_{0}}{b_{0}})k_{eq}+k_{cell}}\right)}^{\frac{1}{3}}$$ (7)

$$b=\frac{3V_{cell}}{4\pi a^{2}}$$ (8)

where *a* is the axis parallel to the elongation vector, *b* is the axis perpendicular to the elongation vector, *V*_*cell*_ is the volume of the cell, *k*_*cell*_ is the cell’s stiffness, and *k*_*eq*_ is the equilibrium stiffness of the bonds between the cell and the ECM defined by,

$$k_{eq}=\frac{n_{b}k_{I}k_{ecm}}{n_{b}k_{I}+k_{ecm}}$$ (9)

where *n*_*b*_ is the number of bonds between the cell and the ECM, *k*_*I*_ is the bond stiffness, and *k*_*ecm*_ is ECM stiffness. Here we assume the bonds and ECM behave as Hookean springs, with the bonds in parallel with each other and in series with the ECM.

The sphericity of the cell is then used to define where along the cell membrane a new pseudopod will extend, defined by the following equation,

s=π3(6Vcell)23Acell (10)

where *V*_*cell*_ is the volume of the cell and *A*_*cell*_ is the cell’s surface area.

#### Pseudopod extension direction

The pseudopod of a completely spherical cell is assumed to randomly extend anywhere on its surface–as opposed to a limited arc centered about the major axis of a more ellipsoid cell. This arc is determined from the extension angle defined by,

$$\Theta_{ex}=180s^{2}$$ (11)

The extension vector is randomly defined within this region and later used to determine the cell’s choice of fiber. In our model, after retraction the cell selects the fiber that most closely follows its extension vector. We add a bias to simulate polarization of cell, by giving protrusions a 80% chance to occur on the leading edge of the cell to simulate microtubule mediation of pseudopod inhibitory signals in the rear of the cell [24]. A polarized cell will follow the newly selected fiber along the acute angle to its previous direction 80% of the time, whereas a non-polarized cell will only follow the acute angle 50% of the time.

#### Matrix fiber cross-link density

Fiber crosslink density *ρ*_*X*_ is calculated using the fiber alignment and fiber density. The number of intersections per unit length of fiber is assumed to be a function of the fiber alignment and is calculated by,

$$n_{X}=\frac{L_{fiber}\rho_{Xmax}\theta_{f}}{\rho_{Xmax}+4.55}$$ (12)

where *L*_*fiber*_ is the fiber length *ρ*_*Xmax*_ is the maximum number of crosslinks per μm when the fibers are randomly aligned [29]. The fiber crosslink density is then determined by multiplying *n*_*X*_ by the fiber density.

#### Matrix stiffness

Gel stiffness is calculated using the bilinear relationship from Lin *et al*. [29],

$$E_{gel}=\left\{ \begin{array}{c} 1039.9n_{X}-1992.9,\;n_{X}<3.5crosslinks/\mu m\\ 5247.9n_{X}-16274,\;n_{X}\geq 3.5crosslinks/\mu m \end{array}\right.$$ (13)

Stiffness of the extra-cellular matrix *k*_*ecm*_ is then derived by converting gel stiffness to N/μm by multiplying *E*_*gel*_ with a characteristic distance (~ 100 nm to 1 μm which corresponding to the thickness of an extending pseudopod [53,73]).

### Model simulation

The central algorithm underlying our simulation is shown in Fig 9. The random seed for the simulation is set using the simulation’s file name amended with a timestamped suffix. Dependent and independent parameters are initialized as described above, and the cell’s initial position and polarity are set. A random number of local fibers are stochastically generated, and the distance to the next fiber intersection is set. Each of the fibers generated is then checked against the cell’s polarity, and the fiber with the minimum angle with the cell’s extension vector is selected. This fiber is divided into elements of length *dl*, and binding sites are distributed into each element using another Poisson distribution with the mean number of bindings given by Eq 1. At this step, the time until a secondary pseudopod will extend is also set.

The cell then enters the outgrowth phase in search of clusters of adhesion sites along the fiber [27]. In this phase, the cell sequentially examines each element along the fiber and forms bonds with the binding sites located within. Outgrowth will continue as long as a minimum number of stable bonds *ρ*_*min*_ forms along the fiber, but stop if a new pseudopod begins extending or if not enough bonds are formed [9,68]. Should the pseudopod encounter a fiber intersection, a new fiber at a stochastically determined angle is generated and the pseudopod follows the acute angle between the old and the new fiber with a 99% likelihood.

If during the outgrowth phase the cell does not find enough binding sites or a new pseudopod begins extending, the cell enters the retracting phase [9,27]. In this phase, the cell immediately stops searching along its current path and extends a pseudopod in a new direction. To replicate the higher distribution of protrusion inhibitory signals in the rear of the cell [24], the cell is given a 20% chance of reversing its direction based on the cell’s polarity any time it retracts an existing pseudopod. New fibers are generated around the cell and a new fiber is selected using the rules described above. The cell will then return to the outgrowth phase as the new pseudopod begins extending.

The pseudopod can only contract once it finds a cluster of binding sites greater than *ρ*_*max*_ and forming a stable bond strong enough to withstand the acto-myosin contractile force [8]. The effective force that the cell experiences is calculated assuming the cell protrusion and the extra-cellular matrix (ECM) fiber network behave as Hookean springs, which is a standard model for force generation by pseudopods [7,74]. For a given cell protrusion of length *l*_0_ and a protrusion spring constant *k*_*cell*_, the force needed to maintain connection to the ECM can be written as,

$$F_{0}=k_{cell}l_{0}$$ (14)

We assume *F*_0_ is dependent on the number of bonds *n*_*b*_ formed between the pseudopod and the ECM and can be described as a simple hill equation type function with coefficient 1. This relationship can be expressed as,

$$F_{0}=\frac{F_{max}\sum_{l_{0}}n_{b}}{\sum_{l_{0}}n_{b}+n_{1/2}}$$ (15)

This is a standard assumption that has been used previously to describe bond density dependent force generation in pseudopods [55,74]. Here *n*_1/2_ is the number of bonds at which the pseudopod generates half its maximum possible force *F*_*max*_. Because this force and the pseudopod length are known, the spring constant for the protrusion can be written as,

$$k_{cell}=\frac{F_{0}}{l_{0}}$$ (16)

As the pseudopod extends into the ECM and forms bonds at its tip, it forms a two spring in series system. Assuming that the spring constant of the ECM is given by *k*_*ECM*_, the effective spring constant of this two-spring system can be calculated from the following equation,

$$\bar{k}=\frac{k_{cell}k_{ECM}}{k_{cell}+k_{ECM}}$$ (17)

From this, the effective force on the system can be given by,

$$F=\bar{k}l_{0}$$ (18)

Substituting from above, we get the following equation,

$$F=\frac{F_{0}k_{ECM}l_{0}}{F_{0}+k_{ECM}l_{0}}$$ (19)

With this, the instantaneous velocity is calculated by dividing this force by the total friction,

$$v_{i}=\frac{F}{\left(f_{v}+f_{b}\right)}$$ (20)

where *f*_*v*_ and *f*_*b*_ are the drag forces due to viscous friction and bond dissociation [73], respectively. *f*_*v*_ is calculated from the following equations,

$$f_{v}=6\pi \eta bK^{\prime }$$ (21)

where *η* is the viscosity of the ECM, *b* is the equatorial semi-axis of the cell, and *K*′ is a shape factor defined by the Eq 21 for the motion of a prolate ellipsoid along the semi-axis,

K′=43(β2−1)(2β2−1)(β2−1)12ln[β+(β2−1)12]−β(β=ba) (22)

and Eq 22 for the motion of an oblate ellipsoid,

K′=43(β2−1)β(β2−1)(β2−1)12tan−1[(β2−1)12]+β(β=ab) (23)

Drag forces due to bond dissociation is calculated by,

$$f_{b}=n_{br}\frac{k_{ECM}k_{I}}{(k_{ECM}+k_{I})k_{off}}exp(\frac{-{kt}_{eff}k_{I}}{n_{br}k_{B}T})$$ (24)

$${kt}_{eff}=\frac{F_{0}{k_{ECM}}^{2}{l_{0}}^{3}}{{\left({F_{0}k_{ECM}l}_{0}\right)}^{2}}$$ (25)

where *n*_*br*_ is the number of bonds at the rear of the cell, *k*_*off*_ is the bond dissociation rate, *k*_*I*_ is the stiffness of each individual bond, and *kt*_*eff*_ is the effective equivalent of temperature *T* times the Boltzmann constant *k*_*B*_. The distance the cell moves during each time step *dt* is then calculated by,

$$d=dt\frac{\int_{0}^{l}vdl}{\int_{0}^{l}dl}$$ (26)

The cell’s x, y, and z positions are logged at every 2 second time steps. Once the simulation is completed, we analyze the random walk characteristics of the cell trajectories for a given set of initial conditions, and calculate cell speed, path persistence, and root mean squared displacement. The average speed is calculated by fitting a line to the distance traveled vs time computed at 10 equally spaced time points over the entire simulation time. This velocity includes all the phases of cell migration including outgrowth and retraction which are non-motile phases and is the true measure equivalent to the experimentally measured cell migration speed [21].

#### Persistence calculation

Path persistence length *L*_*P*_ is determined by using a non-linear least squares regression to fit a nonlinear curve to the following equation;

$$\langle R^{2}\rangle =2{L_{P}}^{2}(\frac{L}{L_{P}}-1+e^{-\frac{L}{L_{P}}})$$ (27)

〈*R*^2^〉 is calculated by averaging the squared displacement between the ends of non-overlapping contours of length *L* that make up the total path. This value is then plotted against increasing contour lengths *L* up to 60 μm in length. The maximum length is fixed in order to eliminate any variance between slow and fast migrating cells.

#### Mean squared displacement

Mean squared displacement is used to determine the random motility coefficient μ and exponent α by fitting the trajectory data to the following equation;

$$\langle R^{2}\rangle ={\mu \tau }^{\alpha }$$ (28)

where *R* is the displacement between time *t* and t + τ. As with *L*_*p*_, 〈*R*^2^〉 is calculated using non-overlapping segments for the given lag time τ [20]. Mean squared displacements are calculated for lag times up to 1/8^th^ of the simulation time. The goodness of fit for these values is then validated by obtaining the coefficient of determination r^2^ using MATLAB’s curve fitting toolbox.

#### Simulating cell mediated matrix degradation

We can incorporate cell mediated degradation by locally updating the ECM stiffness in a region of space that a cell has passed through. We indirectly simulate cell-cell interactions by accounting for how cells degrade the extracellular matrix they pass through and alter its stiffness by cleaving the crosslinks between fibers. We assume the number of crosslinks broken is constant and proportional to the number of cells that passed through a given volume of the ECM (V = 25x25x25 μm^3^). At the start of the contraction phase for a given cell, we calculate the new number of crosslinks with the following equation,

$$n_{X}=2.09+\frac{2.439}{{0.2N}_{cells}+1}$$ (29)

where 2.09 is the minimum number of possible crosslinks and 4.529 is the maximum to represent the range given by Lin *et al* [29]. *N*_*cells*_ is the number of cells that pass through the volume V around the cell. We then use this new value of *n*_*X*_ to determine the stiffness of the ECM surrounding the cell according to Eq 13 as well as the average distance between the crosslinks along the fibers.

#### Cluster analysis of motility phenotypes

In order to analyze the combined influence of multiple parameters on cell motility, we perform a cluster analysis to classify states of high and low motility. The parameters selected in this analysis are fiber alignment, fiber density, and fiber diameter. We simulate 3,000 individual cells, with the value of their initial input parameters randomly chosen from within its physiological range. Cell migration for each input parameter set is repeated 10 times, giving a total of 30,000 simulations. Values for cell speed and persistence length are calculated in the same way as the individual parameter simulations and multiplied (*vL*_*p*_) to obtain an effective diffusion coefficient for the migrating cells.

In order to classify the cell migration data into high and low motility phenotypes we use the top and bottom 15^th^ percentile of calculated diffusion coefficients. We use this analysis to determine the shortest route to altering cell motility phenotype from the high to the low motility. This is done by identifying the line joining the centroids of the two motility phenotype clusters. Motility phenotype clusters and their centroids are obtained using a standard k-means clustering algorithm. The line connecting the centroids between clusters of high and low diffusion is then used to indicate the fastest path between the two states. As this is a binary classification, this path indicates the discrete changes that should be made to either enhance or inhibit cell migration, rather than a continuous pathway that will allow for modulated influence.

## Supporting information

S1 Fig Convergence analysis for simulation time. **A)** Simulation time vs. cell speed **B)** Simulation time vs. persistence length **C)** Simulation time vs. r^2^ for velocity prediction of fast and slow-moving cells. **D)** Simulation time vs. r^2^ for persistence length prediction of fast and slow-moving cells. ρ_i_ = 5.2 sites/monomer for 5 μm/hr, ρ_i_ = 5.75 sites/monomer for 45 μm/hr, and ρ_i_ = 7 sites/monomer for 9 μm/hr. C_gel_ = 3.7 mg/ml, ρ_fiber_ = 1.0 x 10^−3^ fibers/μm^3^, AI = 0, and t_search_ = 16s for all simulations. n = 20. Error bars represent ± SEM. Smoothing splines added to emphasize trends.
(TIF)

S2 Fig Convergence and curve fitting analysis for time step. **A)** Time step vs. cell speed **B)** Time step vs. persistence length **C)** Time step vs. random motility coefficient **D)** Time step vs. r^2^ for cell speed prediction **E)** Time step vs. r^2^ for persistence length prediction **F)** Time step vs. r^2^ for MSD. ρ_i_ = 6 sites/monomer, C_gel_ = 3.7 mg/ml, ρ_fiber_ = 1.0 x 10^−3^ fibers/μm^3^, AI = 0, and t_search_ = 16s for all simulations. n = 20. Error bars represent ± SEM. Smoothing splines added to emphasize trends.
(TIF)

S3 Fig Algorithm efficiency. Time to simulate cell migration vs. simulated time and number of cells. **A)** Time to simulate a single cell. **B)** Time to simulate a given number of cells at 12 h, 24 h, and 48 h. 12hrs is shown in blue, 24 h is shown in red, and 48 is shown in green.
(TIF)

S4 Fig Binding site density vs. time spent in each phase. Blue line is retracting phase, red line is contracting phase, yellow line is outgrowth phase. Optimum migration occurs where time spent in outgrowth and contracting phases is equal.
(TIF)

S5 Fig Trajectories of polarized and nonpolarized cell in aligned matrix. **A)** Blue trajectory is polarized cell, red trajectory is nonpolarized cell. Axes units are in μm. **B)** Comparison of displacement in the direction of fiber alignment vs. time for polarized and nonpolarized cells. **C)** Comparison of average velocity in the direction of fiber alignment vs. time for polarized and nonpolarized cells. Velocity is averaged over 5 minute intervals and then fit with a smoothing spline. AI = 0.8, C_gel_ = 3.7 mg/ml, ρ_i_ = 5.4 sites/monomer, ρ_fiber_ = 1.0 x 10^−3^ fibers/μm^3^, and t_search_ = 16s. Simulation time = 12hrs.
(TIF)

S6 Fig Random motility coefficient and alpha vs. fiber alignment. Plots for μ, and α as a function of increasing alignment index **A)** Random motility coefficient. **b)** Alpha. C_gel_ = 3.7 mg/ml, ρ_i_ = 6 sites/monomer, ρ_fiber_ = 1.0 x 10^−3^ fibers/μm^3^, and t_search_ = 16s. Simulation time = 48hrs. n = 20. Solid blue lines are polarized cells (◼), dashed red lines are nonpolarized cells (●). Error bars represent ± SEM.
(TIF)

S7 Fig Random motility coefficient vs. cell mechanoactivity. C_gel_ = 3.7 mg/ml, ρ_fiber_ = 1.0 x 10^−3^ fibers/μm^3^, and AI = 0. Simulation time = 48hrs. n = 20. Dotted red lines are 5.2 motifs/monomer (◼), solid blue lines are 6 motifs/monomer (●), dashed yellow lines are 8 motifs/monomer (◆). Error bars represent ± SEM.
(TIF)

S1 File Model Optimization for Predication Accuracy and Processing Time. A brief description of how the simulation time step was determined to optimize prediction accuracy and processing time. Additionally, the speed of simulations as a function of the number of different scenarios simulated in parallel is determined.
(DOCX)
