# Correction: A stochastic algorithm for accurately predicting path persistence of cells migrating in 3D matrix environments

**DOI:** 10.1371/journal.pone.0212253

**Published:** 2019-02-07

**Authors:** Benjamin Michael Yeoman, Parag Katira

The following information is missing from the Funding section: Research reported in this manuscript was supported in part by the Army Research Office (https://www.arl.army.mil/) via a grant to PK (W911NF-17-1-0413).

## References

[1] Yeoman BM, Katira P (2018) A stochastic algorithm for accurately predicting path persistence of cells migrating in 3D matrix environments. PLoS ONE 13(11): e0207216 10.1371/journal.pone.0207216 30440015 PMC6237354

