# Supplementary figures and images for: A stochastic algorithm for accurately predicting path persistence of cells migrating in 3D matrix environments
Source: PLoS One. 2018 Nov 15;13(11):e0207216. doi: 10.1371/journal.pone.0207216 (PMC6237354; doi:10.1371/journal.pone.0207216)

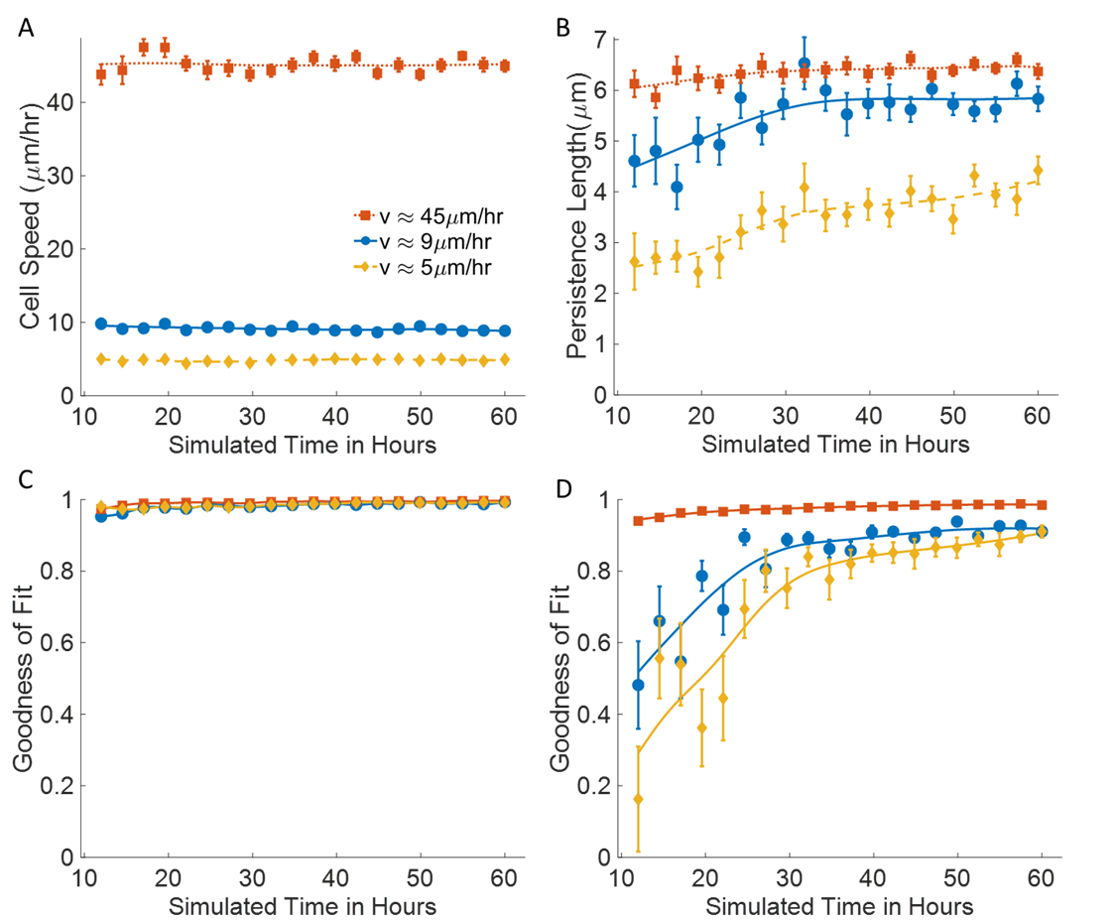

Supplement: S1 Fig — A) Simulation time vs. cell speed B) Simulation time vs. persistence length C) Simulation time vs. r2 for velocity prediction of fast and slow-moving cells. D) Simulation time vs. r2 for persistence length prediction of fast and slow-moving cells. ρi = 5.2 sites/monomer for 5 μm/hr, ρi = 5.75 sites/monomer for 45 μm/hr, and ρi = 7 sites/monomer for 9 μm/hr. Cgel = 3.7 mg/ml, ρfiber = 1.0 x 10^−3^ fibers/μm3, AI = 0, and tsearch = 16s for all simulations. n = 20. Error bars represent ± SEM. Smoothing splines added to emphasize trends. (TIF) [file pone.0207216.s001.tif]

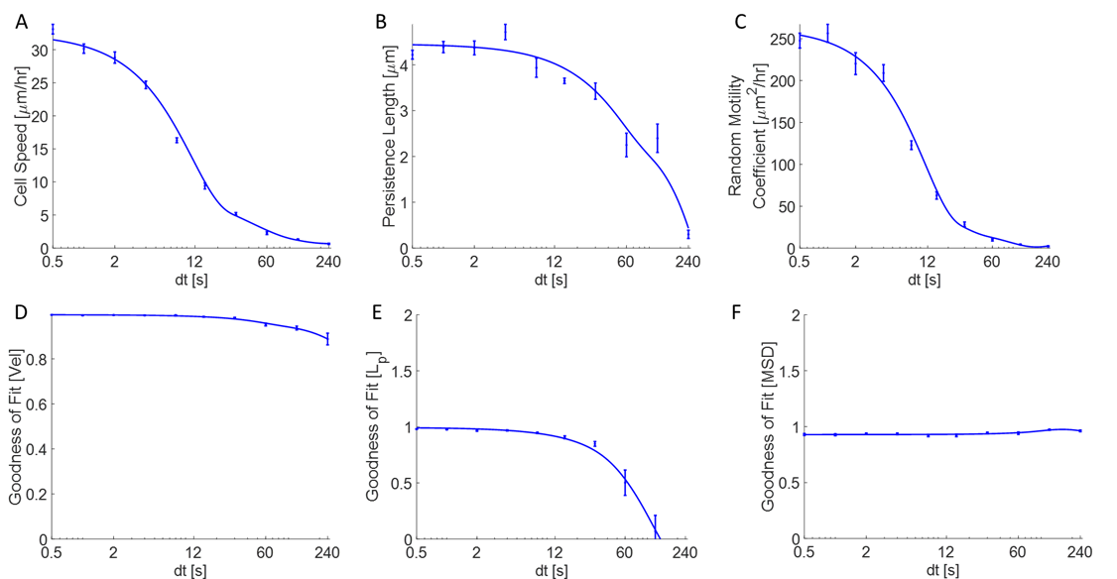

Supplement: S2 Fig — A) Time step vs. cell speed B) Time step vs. persistence length C) Time step vs. random motility coefficient D) Time step vs. r2 for cell speed prediction E) Time step vs. r2 for persistence length prediction F) Time step vs. r2 for MSD. ρi = 6 sites/monomer, Cgel = 3.7 mg/ml, ρfiber = 1.0 x 10^−3^ fibers/μm3, AI = 0, and tsearch = 16s for all simulations. n = 20. Error bars represent ± SEM. Smoothing splines added to emphasize trends. (TIF) [file pone.0207216.s002.tif]

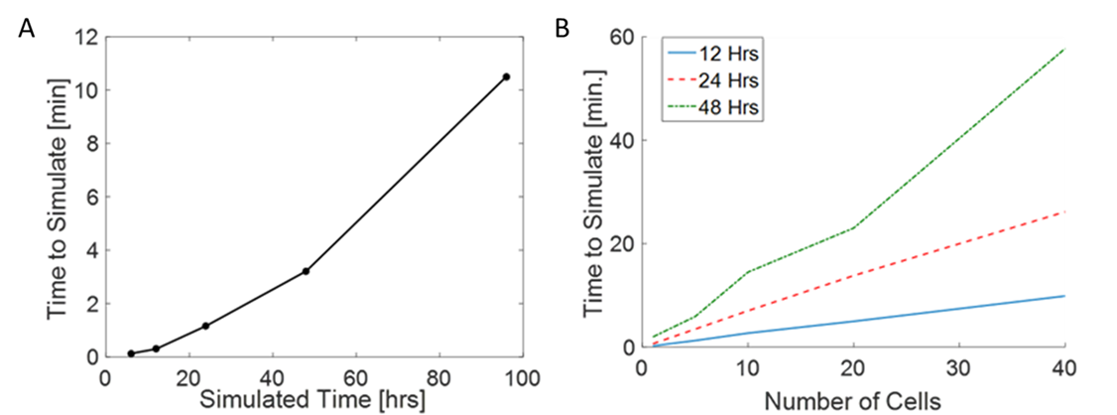

Supplement: S3 Fig — Time to simulate cell migration vs. simulated time and number of cells. A) Time to simulate a single cell. B) Time to simulate a given number of cells at 12 h, 24 h, and 48 h. 12hrs is shown in blue, 24 h is shown in red, and 48 is shown in green. (TIF) [file pone.0207216.s003.tif]

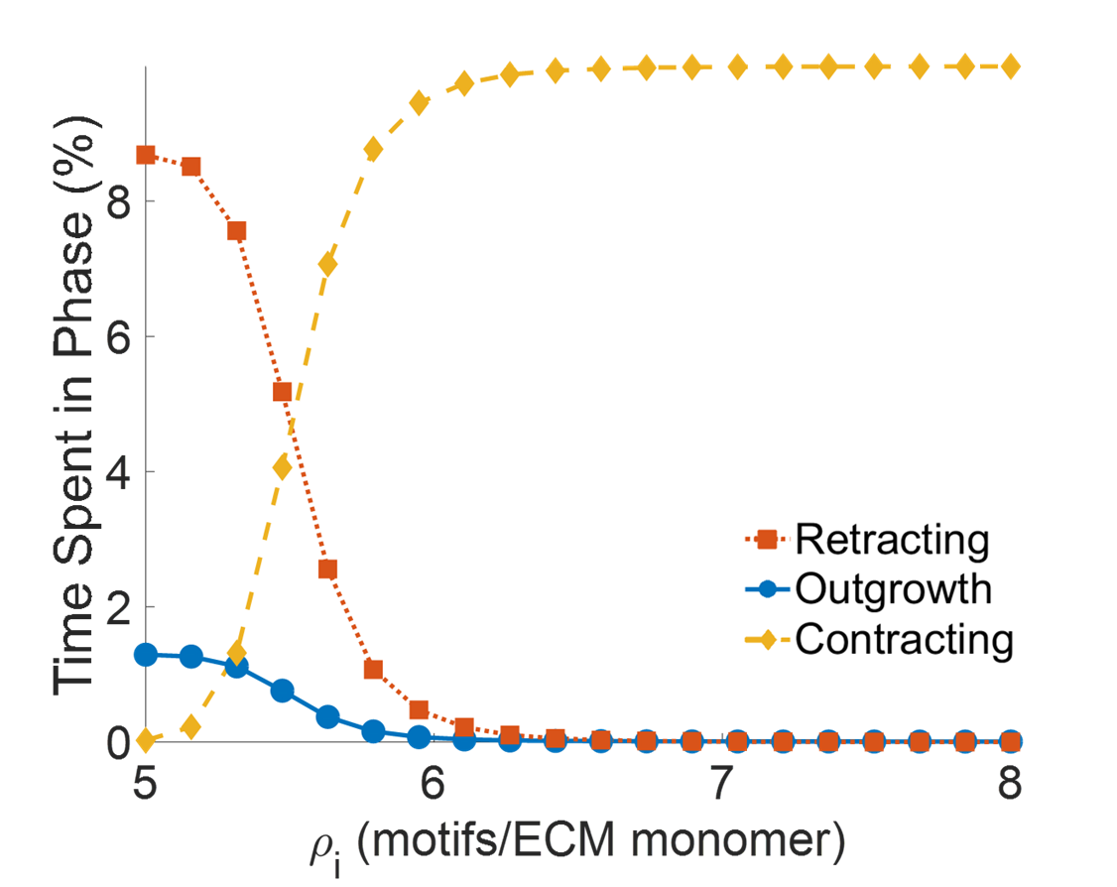

Supplement: S4 Fig — Blue line is retracting phase, red line is contracting phase, yellow line is outgrowth phase. Optimum migration occurs where time spent in outgrowth and contracting phases is equal. (TIF) [file pone.0207216.s004.tif]

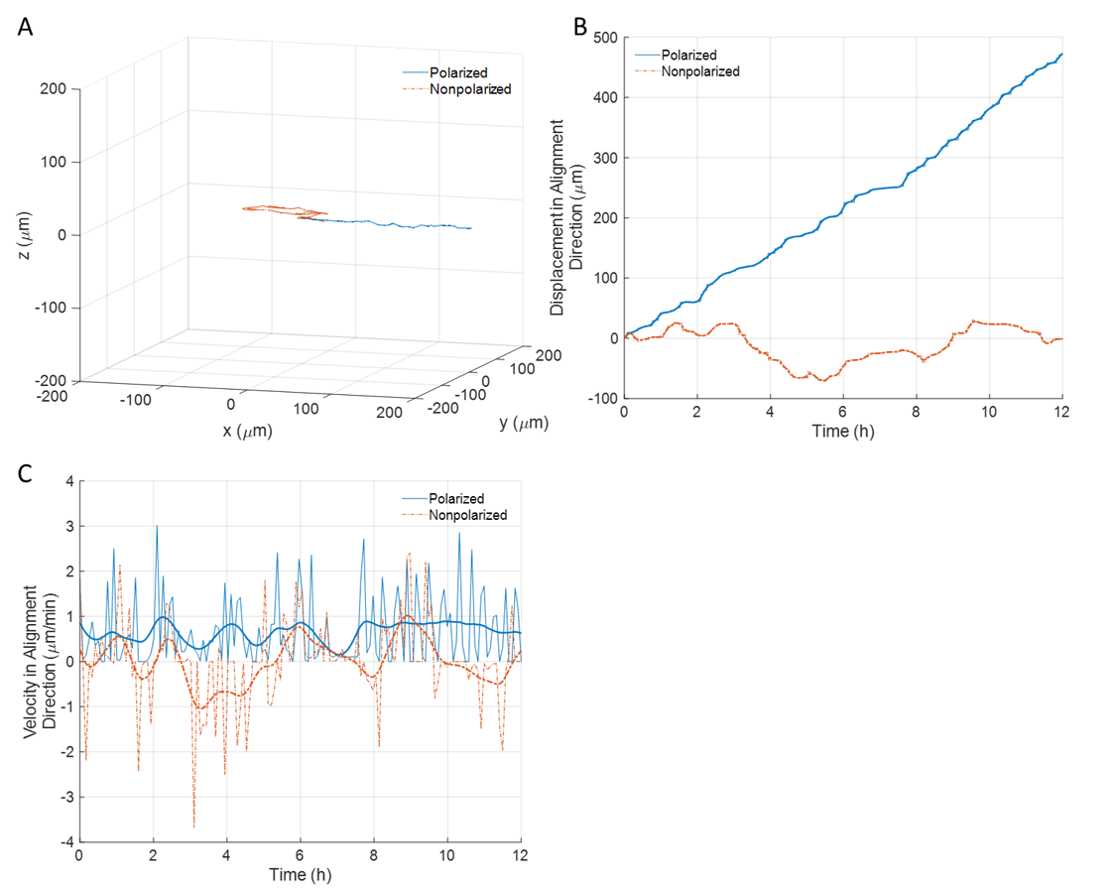

Supplement: S5 Fig — A) Blue trajectory is polarized cell, red trajectory is nonpolarized cell. Axes units are in μm. B) Comparison of displacement in the direction of fiber alignment vs. time for polarized and nonpolarized cells. C) Comparison of average velocity in the direction of fiber alignment vs. time for polarized and nonpolarized cells. Velocity is averaged over 5 minute intervals and then fit with a smoothing spline. AI = 0.8, Cgel = 3.7 mg/ml, ρi = 5.4 sites/monomer, ρfiber = 1.0 x 10^−3^ fibers/μm3, and tsearch = 16s. Simulation time = 12hrs. (TIF) [file pone.0207216.s005.tif]

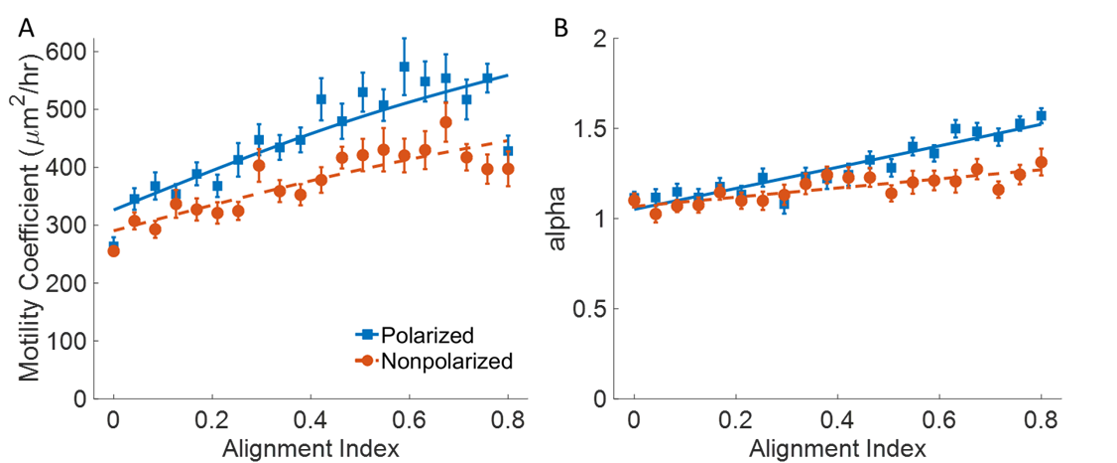

Supplement: S6 Fig — Plots for μ, and α as a function of increasing alignment index A) Random motility coefficient. b) Alpha. Cgel = 3.7 mg/ml, ρi = 6 sites/monomer, ρfiber = 1.0 x 10^−3^ fibers/μm3, and tsearch = 16s. Simulation time = 48hrs. n = 20. Solid blue lines are polarized cells (◼), dashed red lines are nonpolarized cells (●). Error bars represent ± SEM. (TIF) [file pone.0207216.s006.tif]

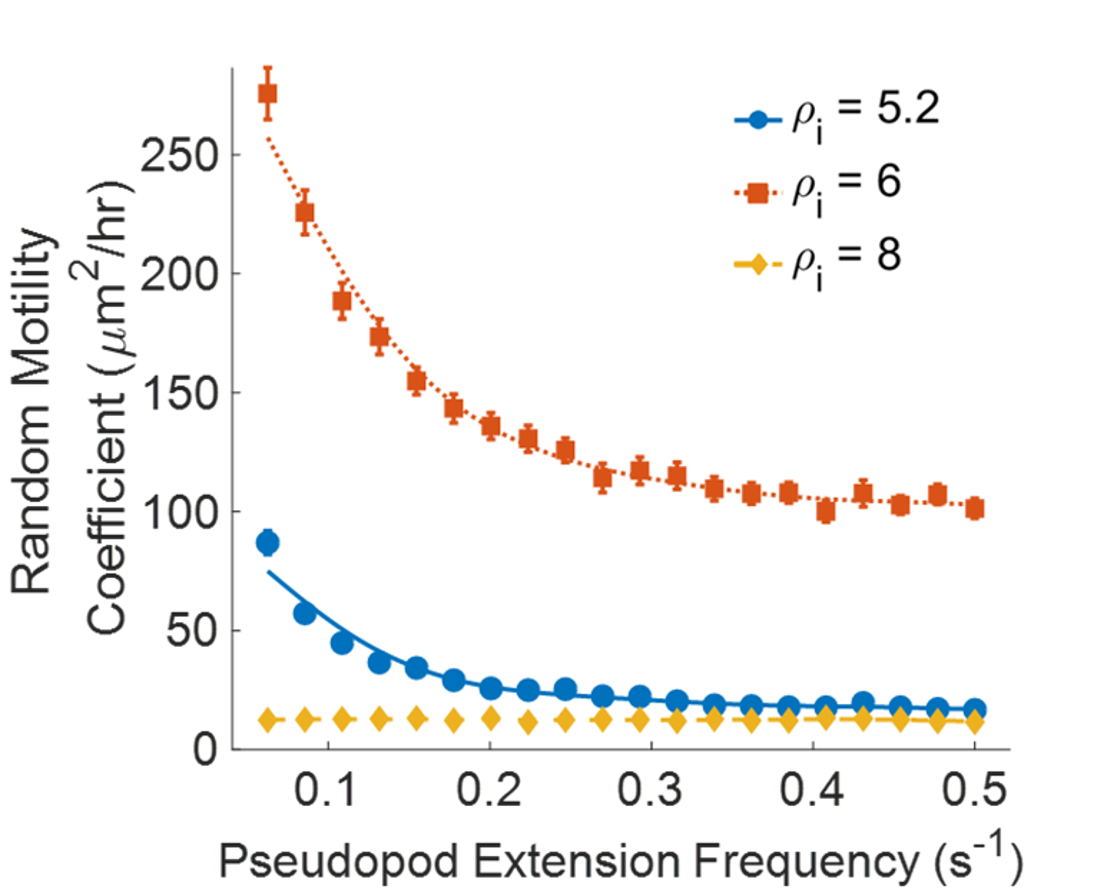

Supplement: S7 Fig — Cgel = 3.7 mg/ml, ρfiber = 1.0 x 10^−3^ fibers/μm3, and AI = 0. Simulation time = 48hrs. n = 20. Dotted red lines are 5.2 motifs/monomer (◼), solid blue lines are 6 motifs/monomer (●), dashed yellow lines are 8 motifs/monomer (◆). Error bars represent ± SEM. (TIF) [file pone.0207216.s007.tif]
