# Supplementary material for: A stochastic algorithm for accurately predicting path persistence of cells migrating in 3D matrix environments
Source: PLoS One. 2018 Nov 15;13(11):e0207216. doi: 10.1371/journal.pone.0207216 (PMC6237354; doi:10.1371/journal.pone.0207216)
Supplement: S1 File — A brief description of how the simulation time step was determined to optimize prediction accuracy and processing time. Additionally, the speed of simulations as a function of the number of different scenarios simulated in parallel is determined. (DOCX) [file pone.0207216.s008.docx]

**Supplemental Information**

**Model Optimization for Predication Accuracy and Processing Time**

Persistence length is determined using a non-linear, least squares regression, and its prediction requires sufficiently long trajectories to provide adequate data points for accurate curve fitting. Before analyzing the effects of each parameter, we first determine the number of hours of cell migration needed for cell speed and persistence to converge on a single value, and to provide a successful curve fit for both fast and slow-moving cells. Convergence and accurate curve fitting are tested using initial cell and matrix conditions that provide cell speeds of approximately 5, 9, and 45 μm/hr. The convergence for all cell speeds occurs immediately after 12 hours, but only occurs after 36 hours for persistence length for cell migrating slower than 9 μm/hr, (S1A-B Figs). Similarly, the curve fitting accuracy for slow and fast-moving cells is shown in S1C-D Figs. To predict the persistence length for slow-moving cells, the simulation time must be at least 48 hours to generate trajectories long enough to obtain an r^2^ value greater than 0.9 (S1C Fig).

S3 Fig shows how long it takes to run the simulation and analyze cell trajectories vs. the simulated time and the number of cells simulated. These results are for simulations run in series on a 64-bit Windows 7 operating system, with an Intel Core i3-2348M 2.30 GHz processor with 12.0 GB of installed memory. S3A Fig shows a polynomial increase in the time required to simulate a single cell trajectory with the model time for which the cell migration is simulated. The actual time may vary slightly from what is shown, depending on the cell’s initial conditions. S3B Fig shows a relatively linear relationship for the number of cells simulated in series vs. the simulation time. Our algorithm also has the capability of being run in parallel on a high-performance computing cluster, which improves the efficiency at a higher number of cells (e.g. ~30,000 cells (~4h computation time)), data not shown.
